# Supplementary material for: A combined approach for single-cell mRNA and intracellular protein expression analysis
Source: Commun Biol. 2021 May 25;4:624. doi: 10.1038/s42003-021-02142-w (PMC8149646; doi:10.1038/s42003-021-02142-w)
Supplement: Supplementary file 2 — Supplementary Information [file 42003_2021_2142_MOESM2_ESM.pdf]

Supplementary figures and tables

Contents

Supplementary figures **2**
Supplementary figure 1 2
Supplementary figure 2 4
Supplementary figure 3 11
Supplementary figure 4 13
Supplementary figure 5 19
Supplementary figure 6 24
Supplementary figure 7 25
Supplementary figure 8 26

Supplementary tables **27**
Supplementary table 1 27
Supplementary table 2 30
Supplementary table 3 31

Supplementary figures

Supplementary figure 1

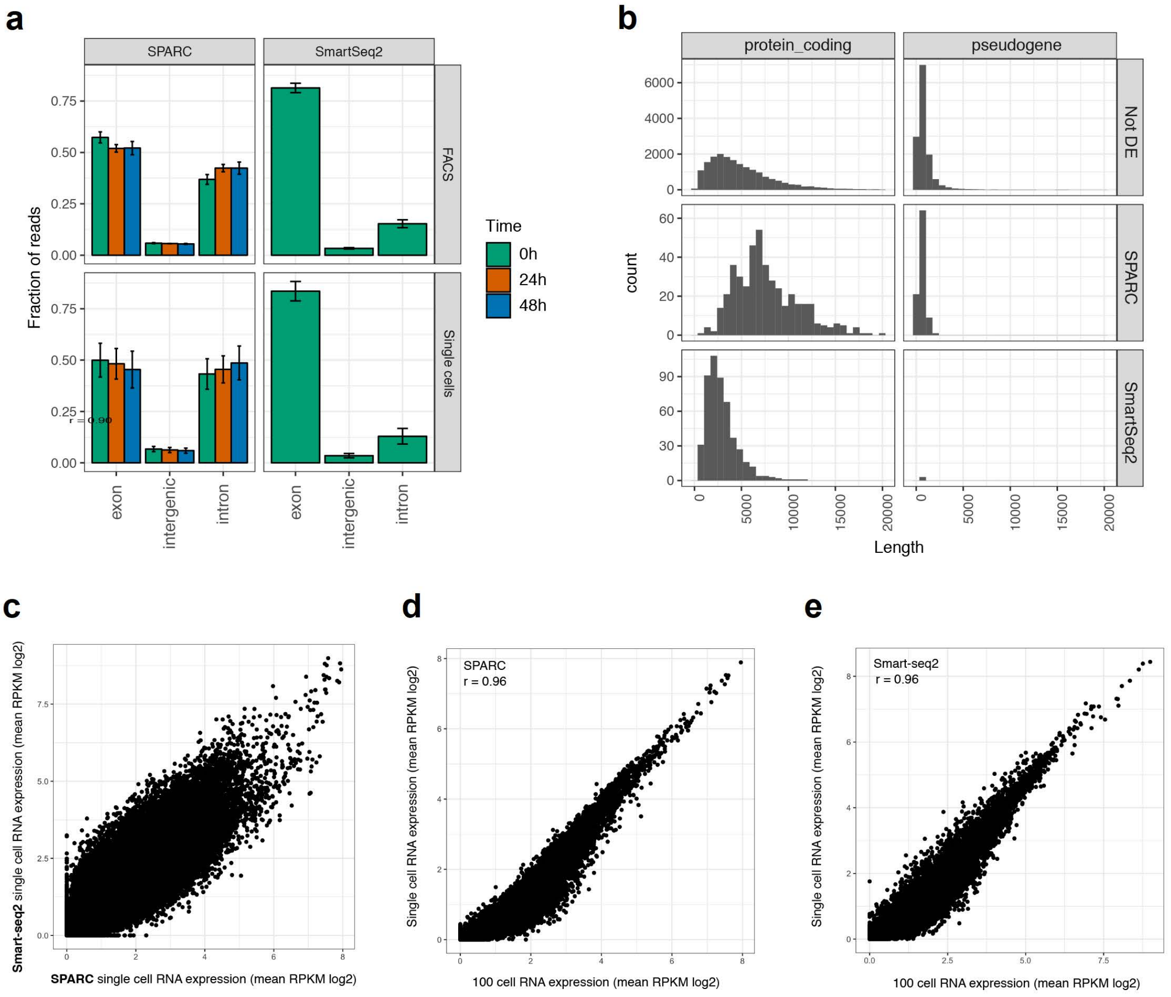

Supplementary Figure 1: Characteristics of SPARC

(a) Comparative characteristics of the single cell mapped sequencing reads (exon, intergenic, intron) for the SPARC versus Smart-seq2 protocol. Data is reported both for the reads originating from the single cell data and the FACS sorted 100 cell control data. **(b)** Annotation of differentially expressed genes detected via SPARC versus Smart-seq2. The genes preferentially detected with SPARC tend to be longer than the genes preferentially detected with Smart-seq2. The Not DE group represent the group of genes equally detected with both methods. **(c)** Single cell mean RKPM (log2) expression for all genes measured using either SPARC (x-axis) or Smart-seq2 (y-axis) (Pearson correlation coefficient $r = 0.90$) **(d)** Comparison of SPARC mean RKPM (log2) RNA expression reads for replicate FACS sorted 100 hESCs at 0h population (x-axis) or single cells (Pearson correlation coefficient $r = 0.96$). **(e)** Comparison of Smart-seq2 mean RKPM (log2) RNA expression reads for replicate FACS sorted 100 hESCs at 0h population (x-axis) or single cells (Pearson correlation coefficient $r = 0.96$).

Supplementary figure 2

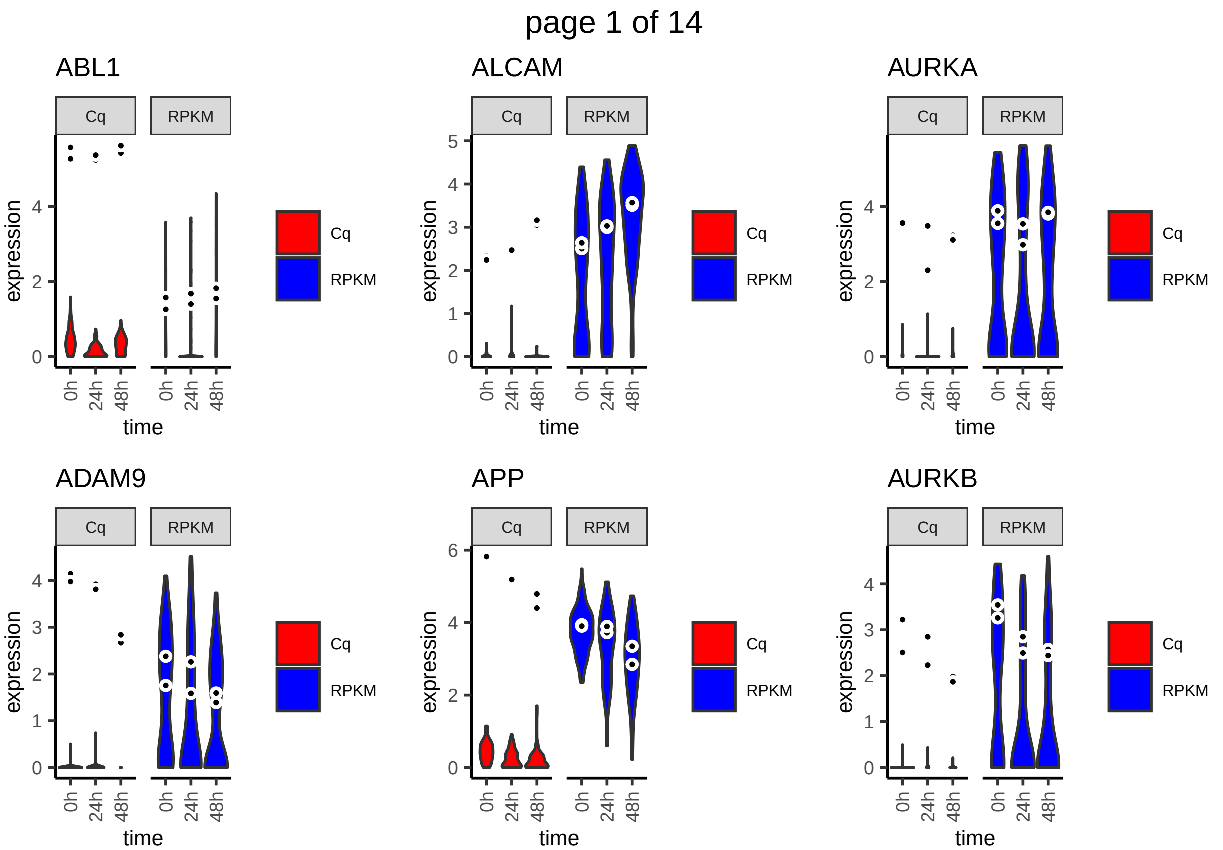

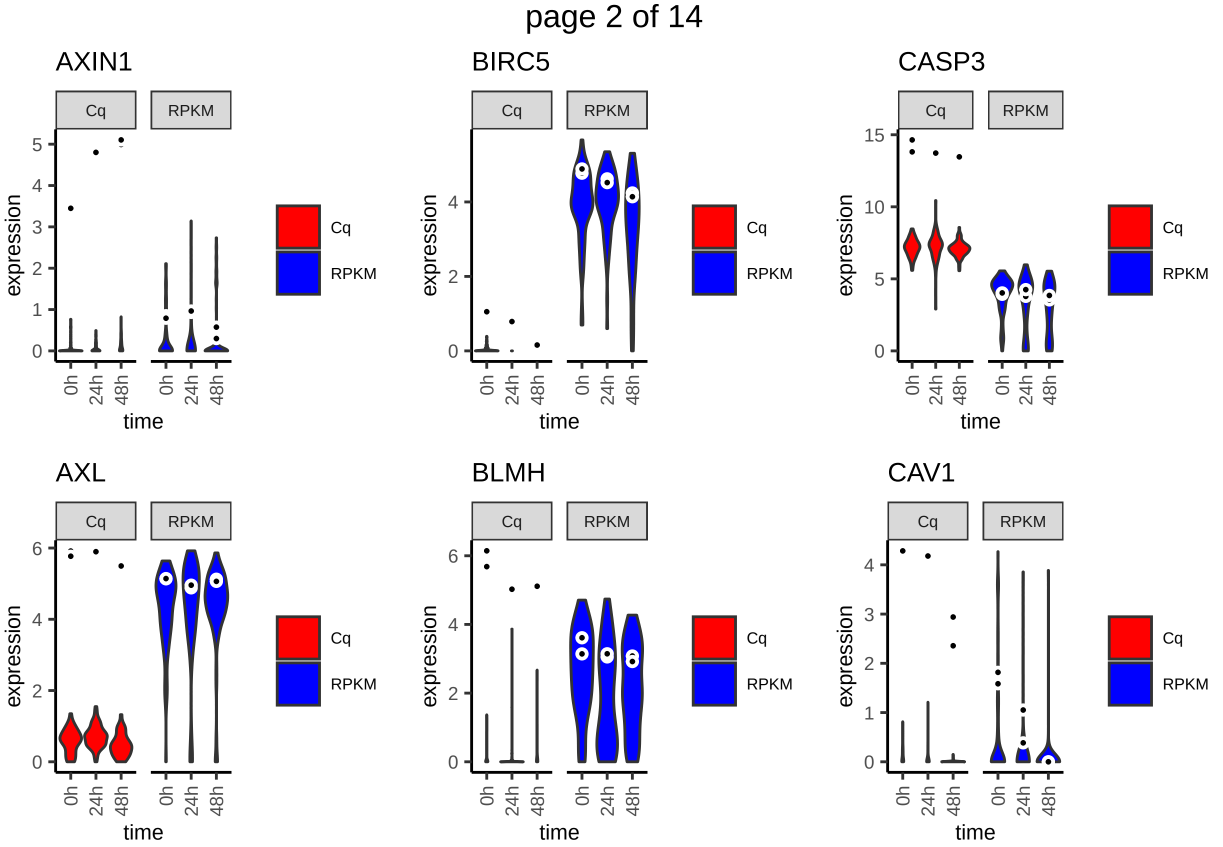

page 3 of 14

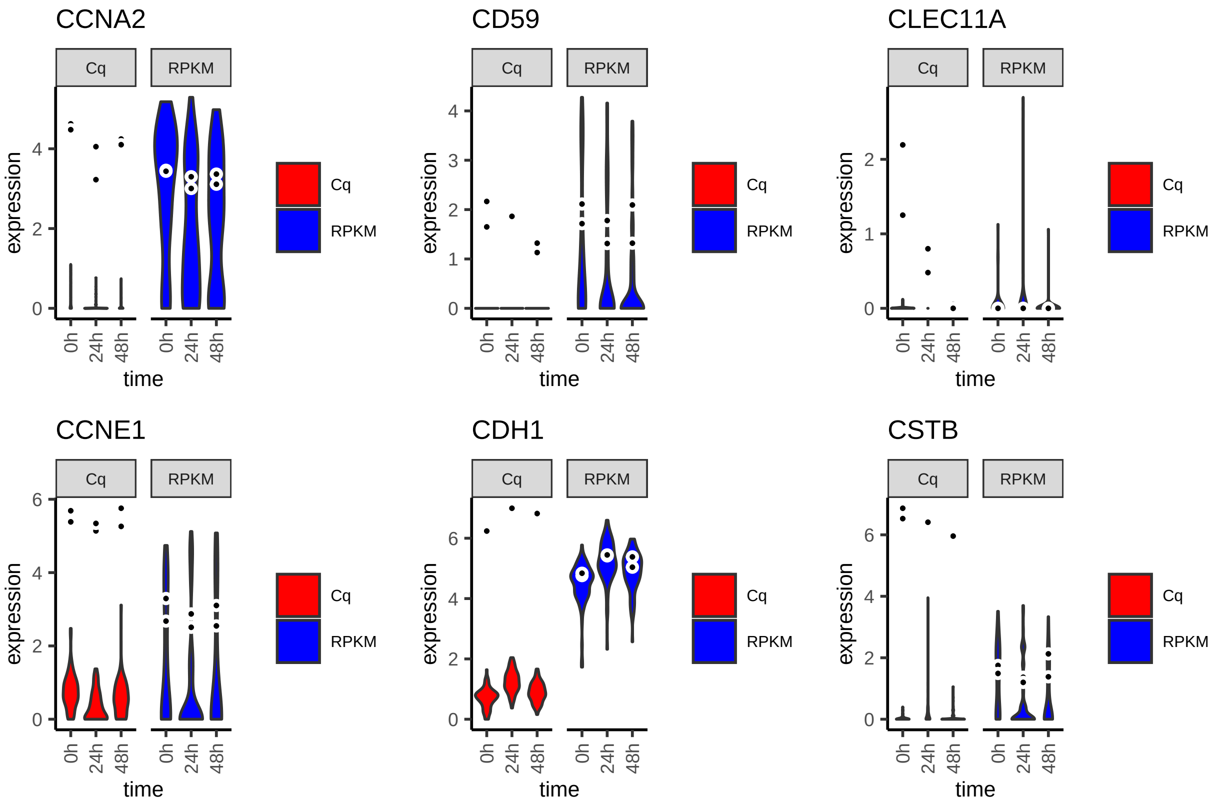

page 4 of 14

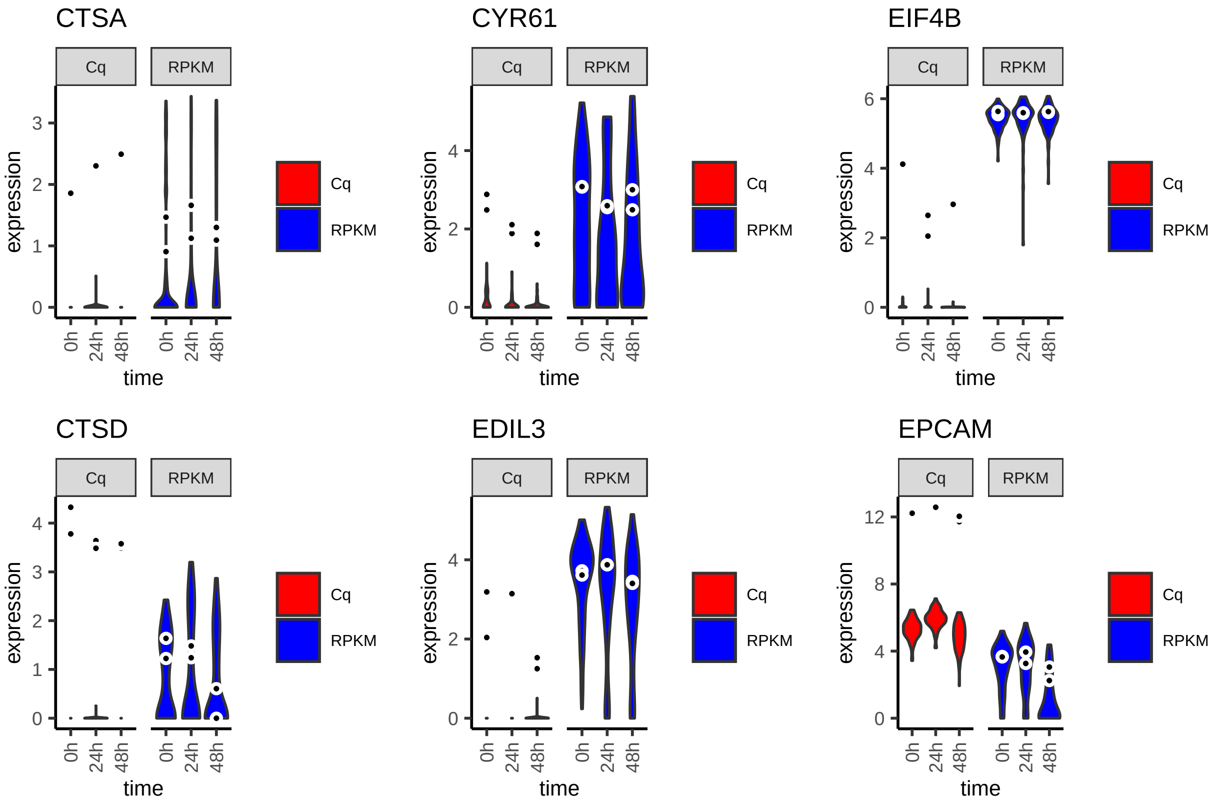

page 5 of 14

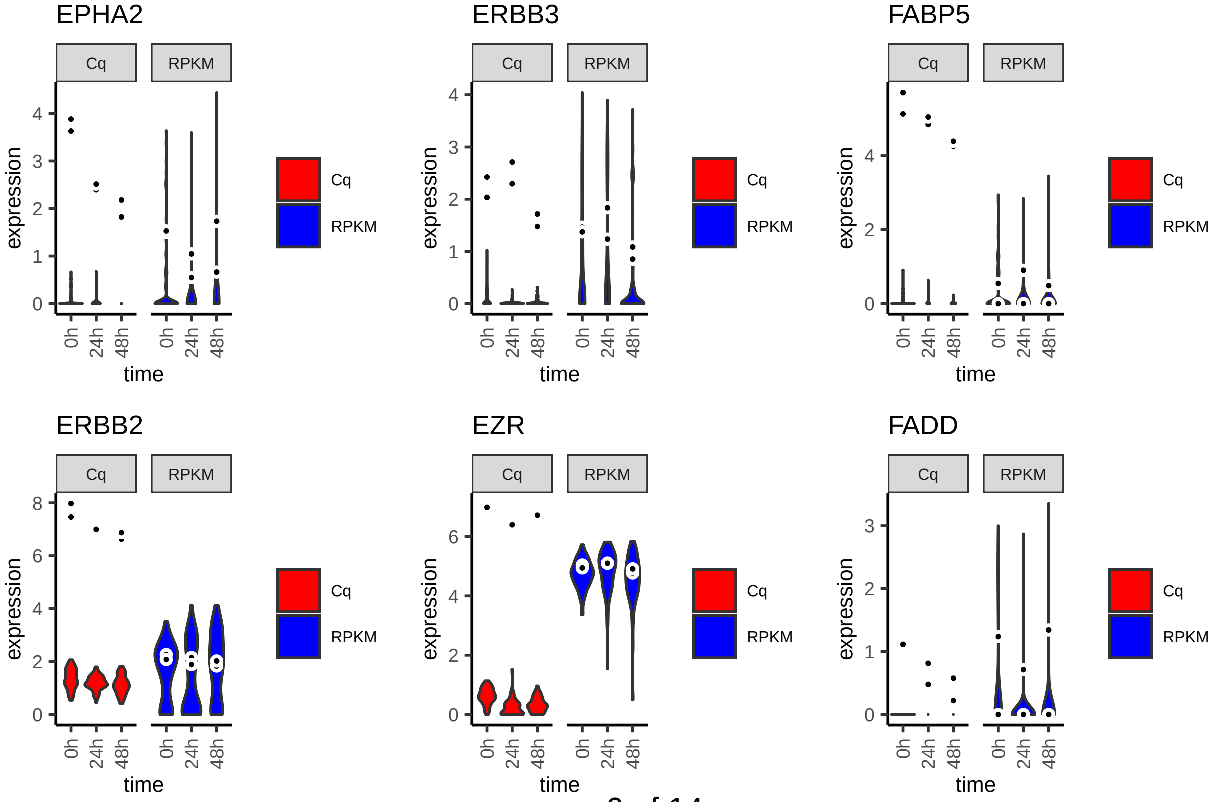

page 6 of 14

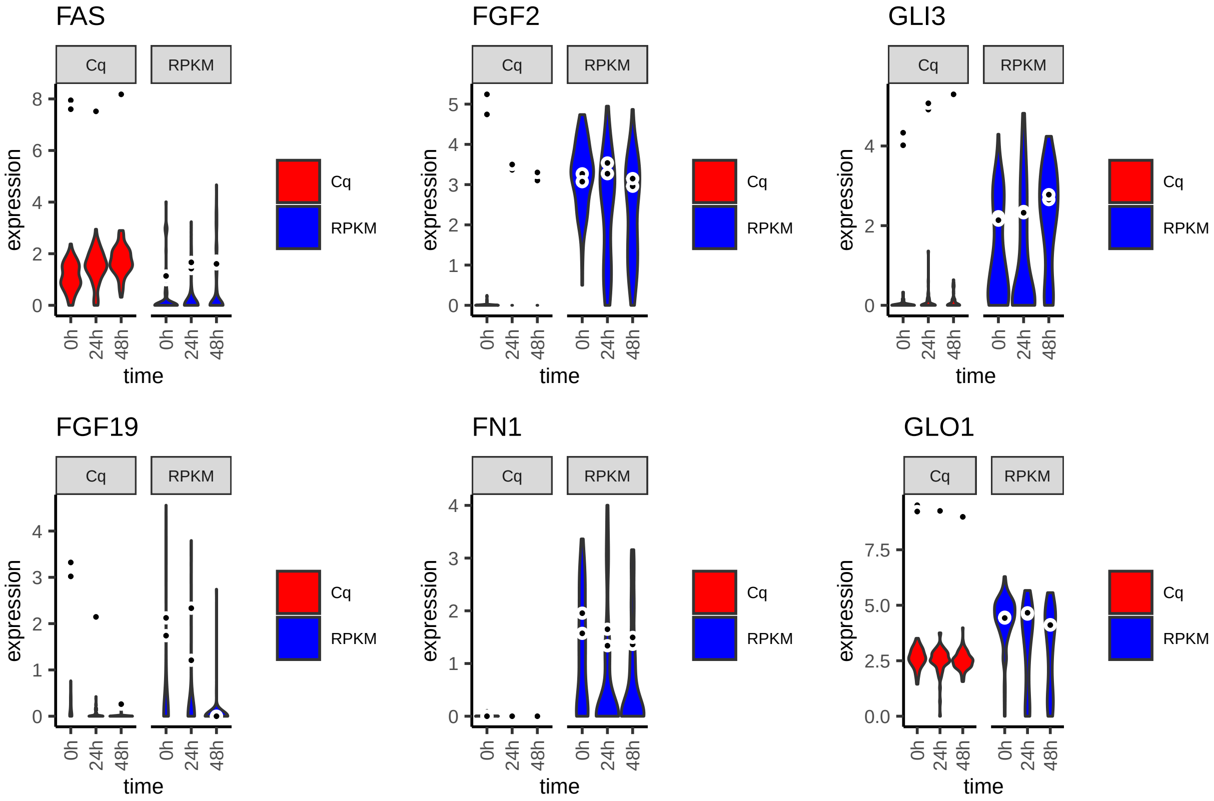

page 7 of 14

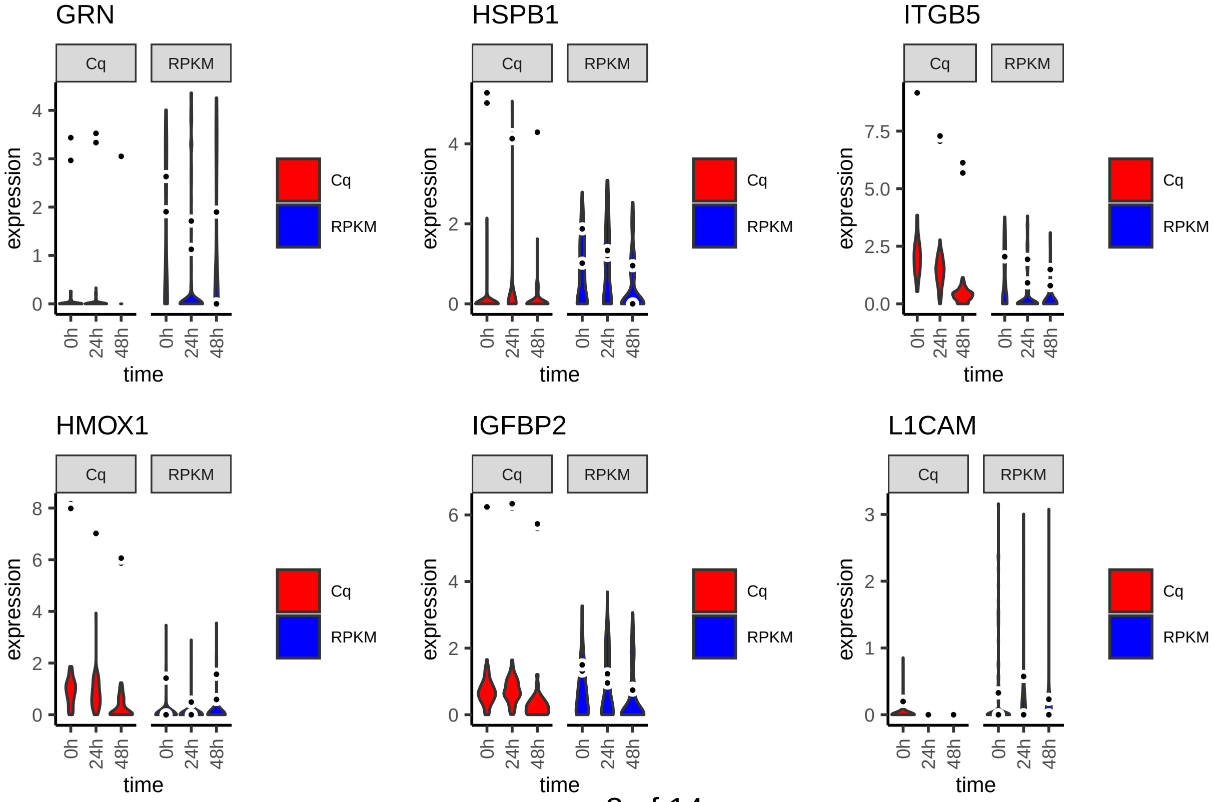

page 8 of 14

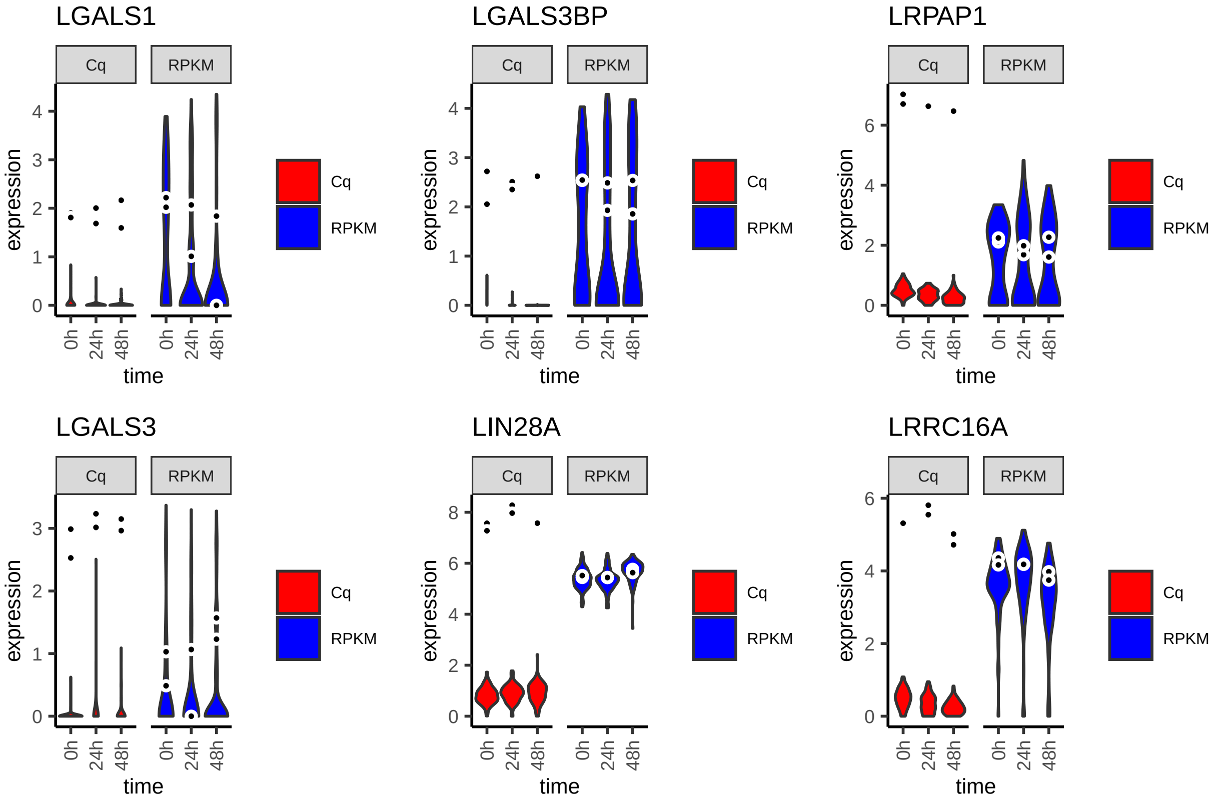

page 9 of 14

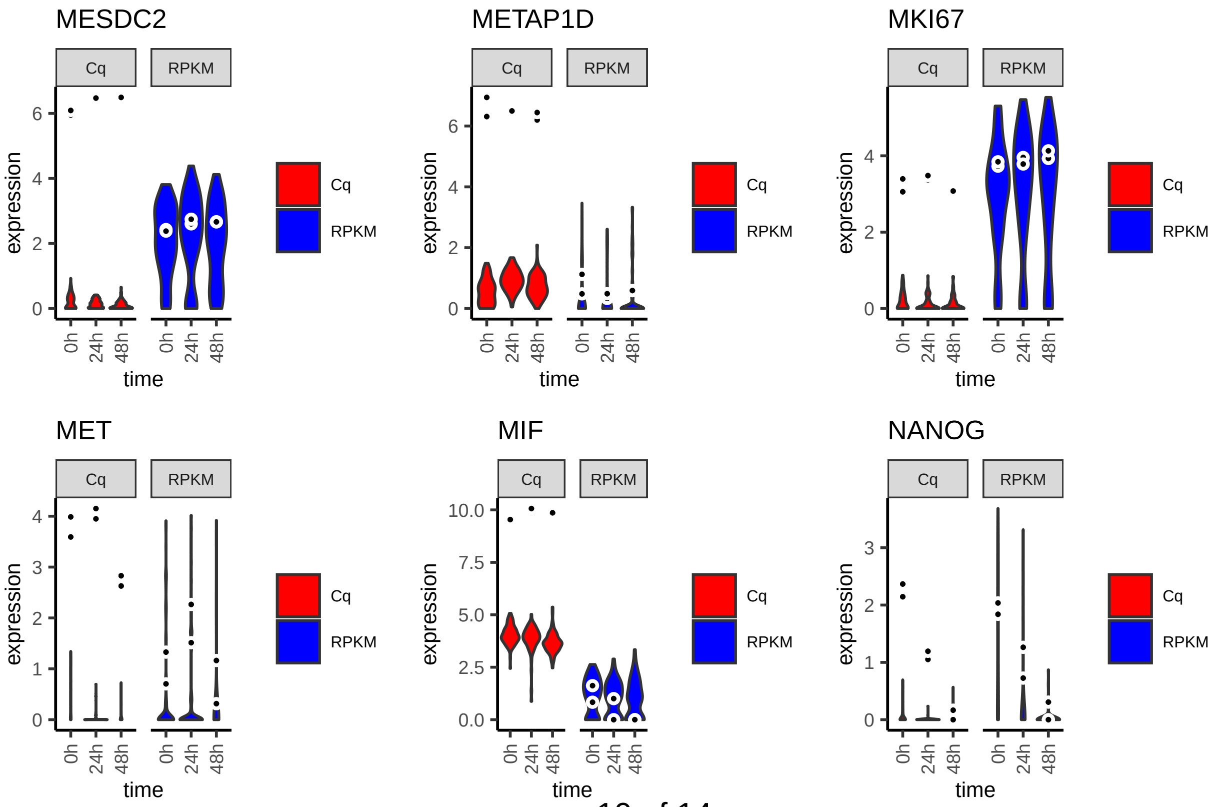

page 10 of 14

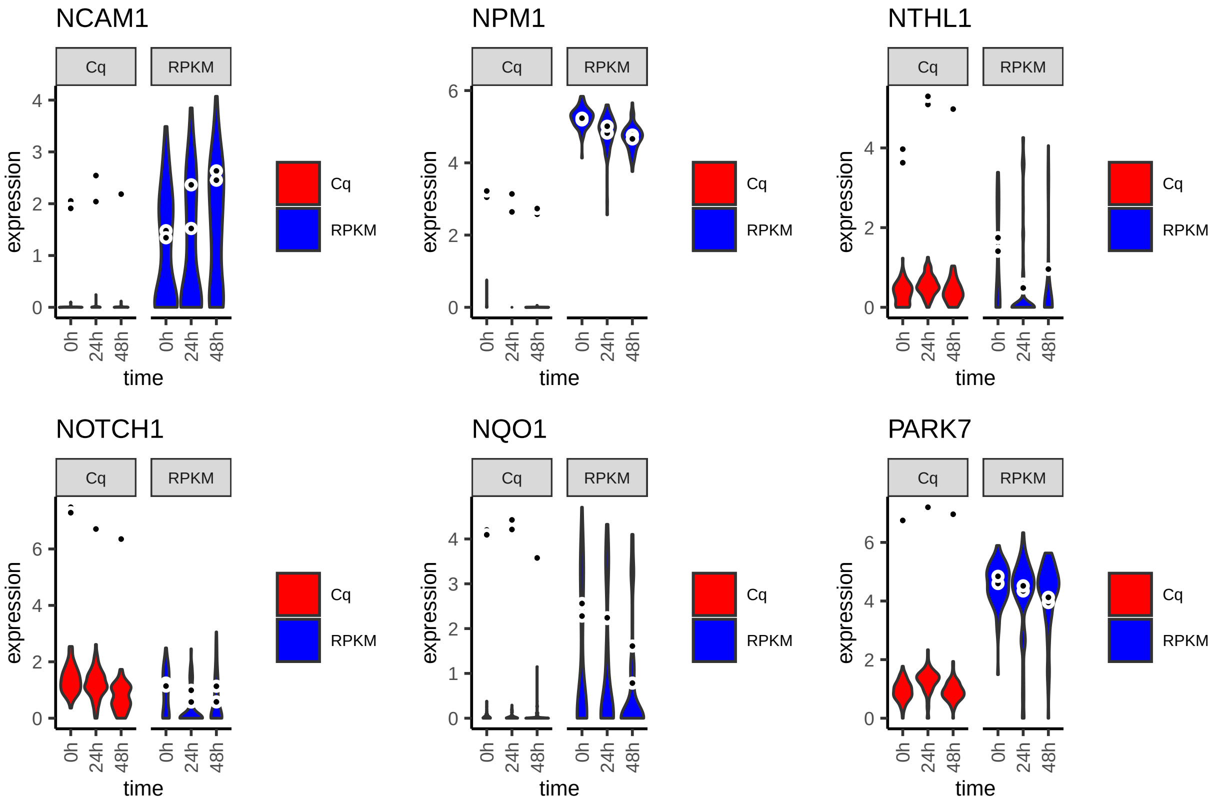

page 11 of 14

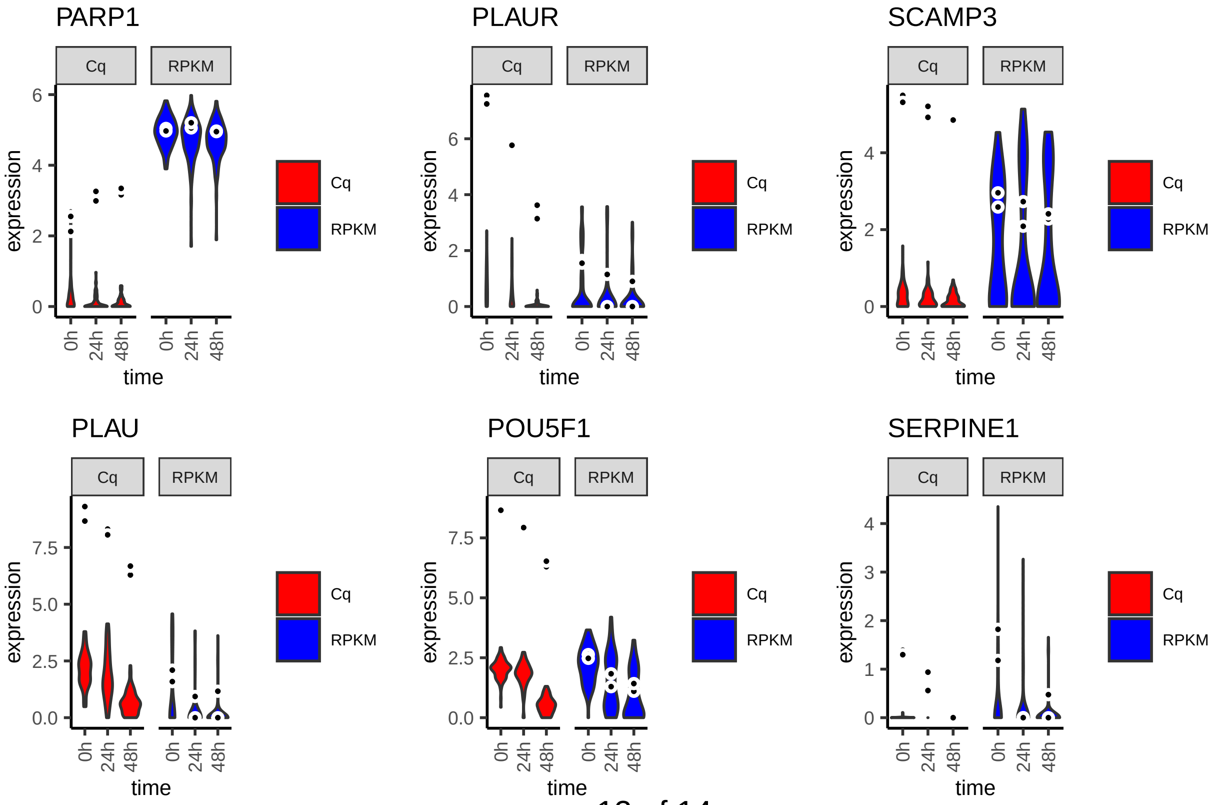

page 12 of 14

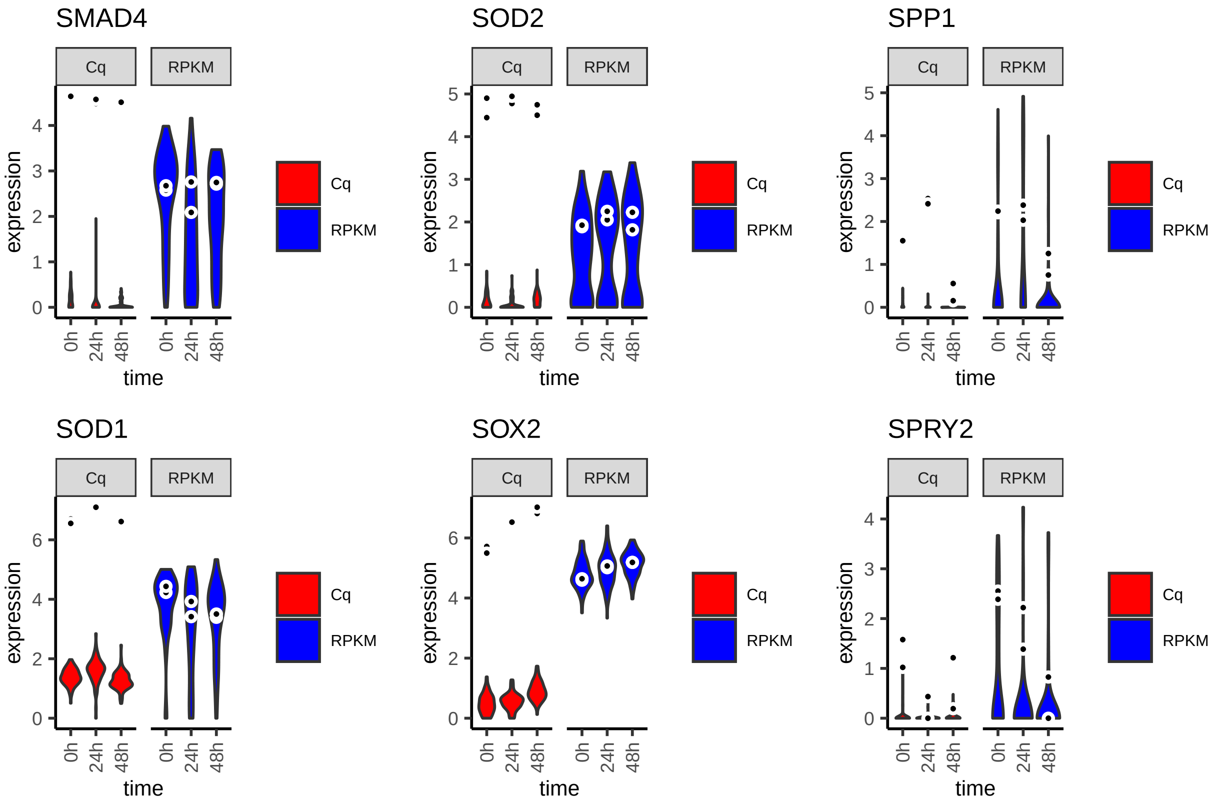

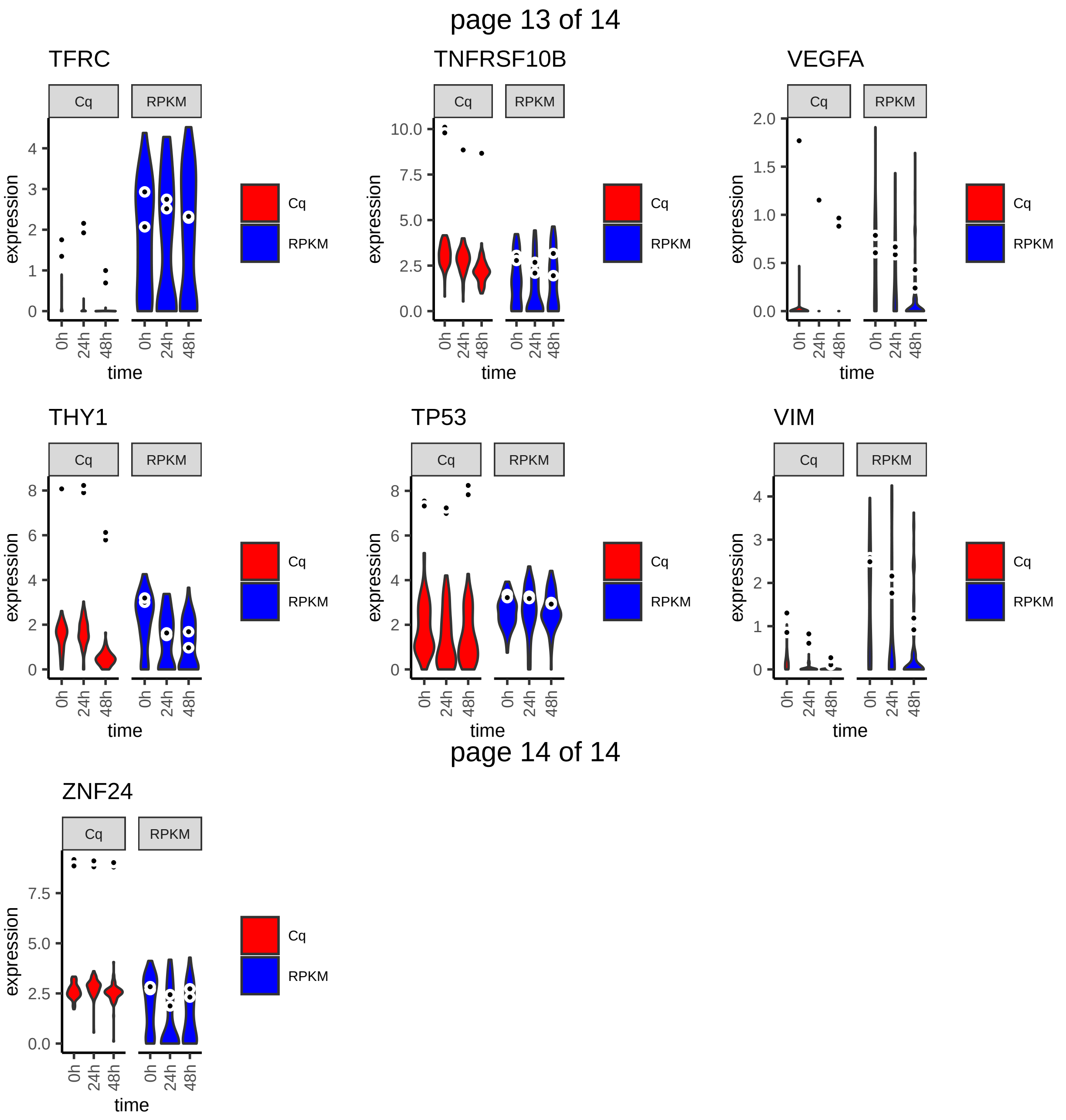

Supplementary Figure 2: RNA and protein gene expression violin plots

Gene expression plots for the genes measured at both the level of protein (red) and RNA (blue). Results are shown for both the duplicate 100 cell control (black dots) and single cells (violin plots). The presented data is not normalized for cell cycle, the number of genes detected (RNA) or cumulative protein sums (protein).

Supplementary figure 3

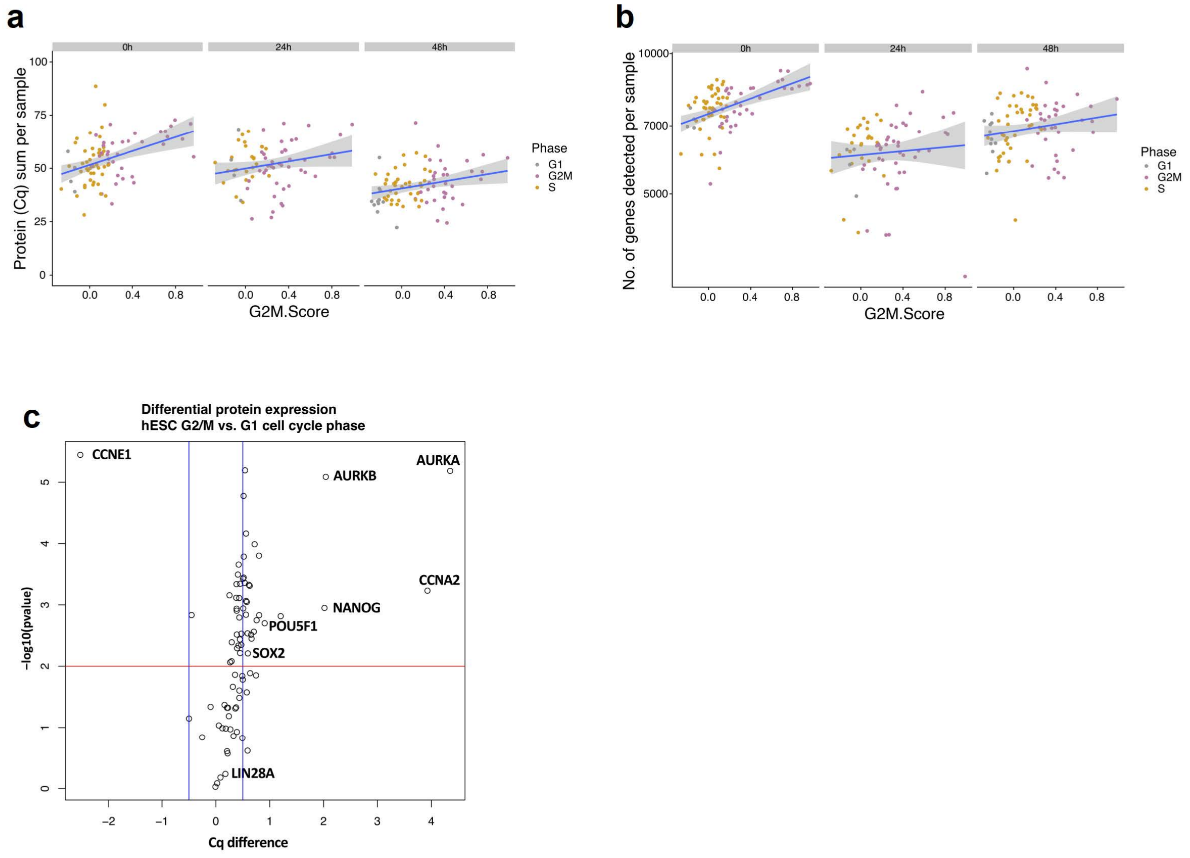

Supplementary Figure 3: Cell cycle and gene expression

(a-b) Relationship between the predicted cell cycle phase (G1, S or G2/M) and **(a)** the per cell cumulative protein sum or **(b)** the number of detected genes. The cumulative protein sum was calculated by summing across all proteins measured (n = 92) per cell. **(c)** hESCs were labeled with the live cell Vybrant DyeCycle Violet DNA stain and sorted by DNA content into G1, S or G2/M phase. 100 cells in triplicate per cell cycle phase were processed for multiplex PEA analysis. Protein expression was compared between G2/M sorted cells and G1 sorted cells. The volcano plot shows the extent of the difference in expression between G2 and G1 cell cycle phases (x-axis) and significance of the difference (y-axis). The red line marks p-value 0.01. The blue lines mark 0.5 Cq difference between the cell cycle phases G2 and G1, or an approximate 1.5-fold difference. As expected, G2-specific proteins AURKA, CCNA2 and AURKB and G1-Gspecific protein CCNE1 show higher expression in G2 and G1 cell cycle phases, respectively. The majority of protein show a mean 1.5 increase in protein amount in G2 versus G1, with some notable exceptions, including e.g. pluripotent factors NANOG and POU5F1.

Supplementary figure 4

page 1 of 9

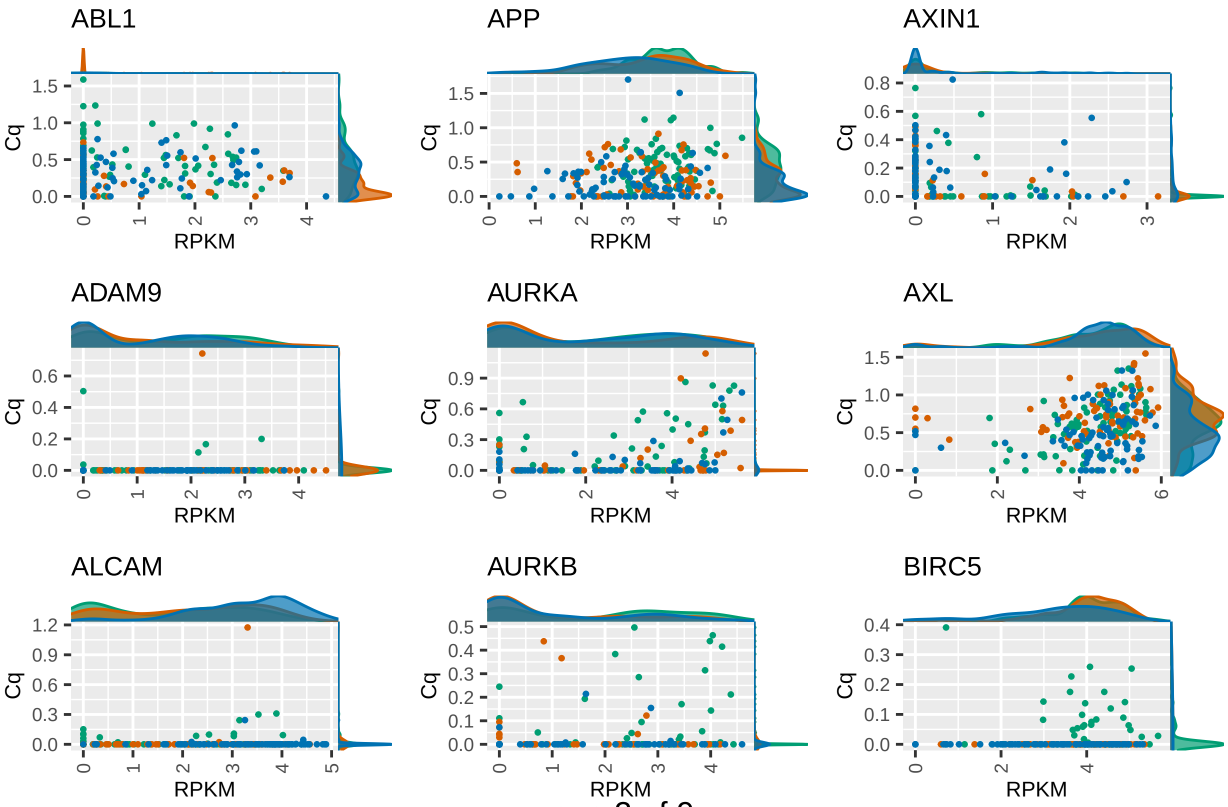

page 2 of 9

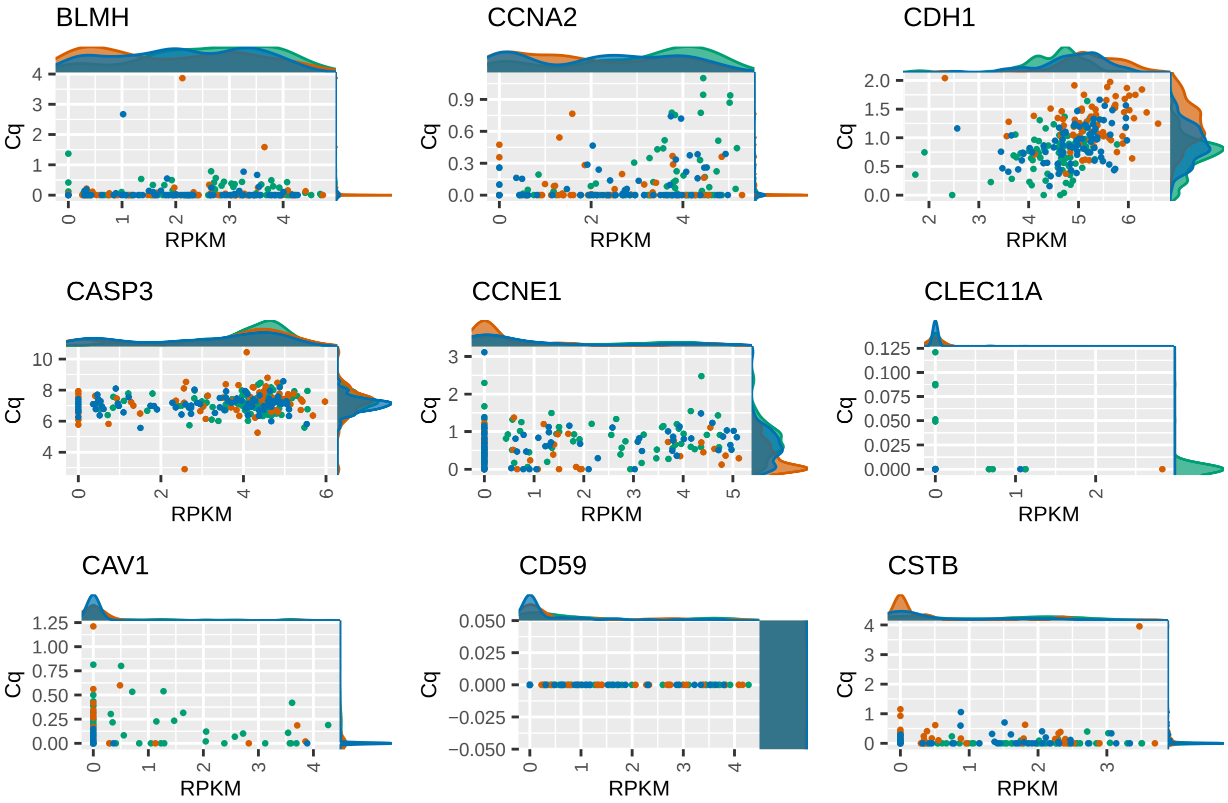

page 3 of 9

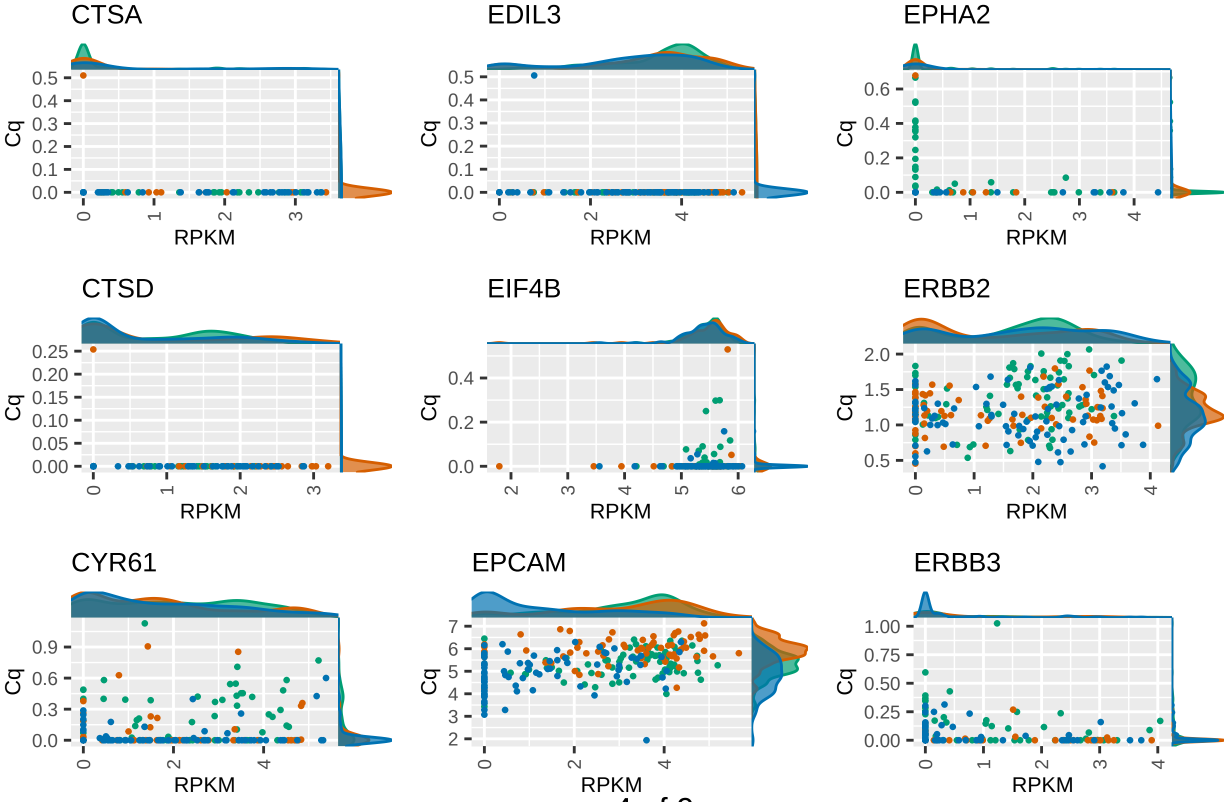

page 4 of 9

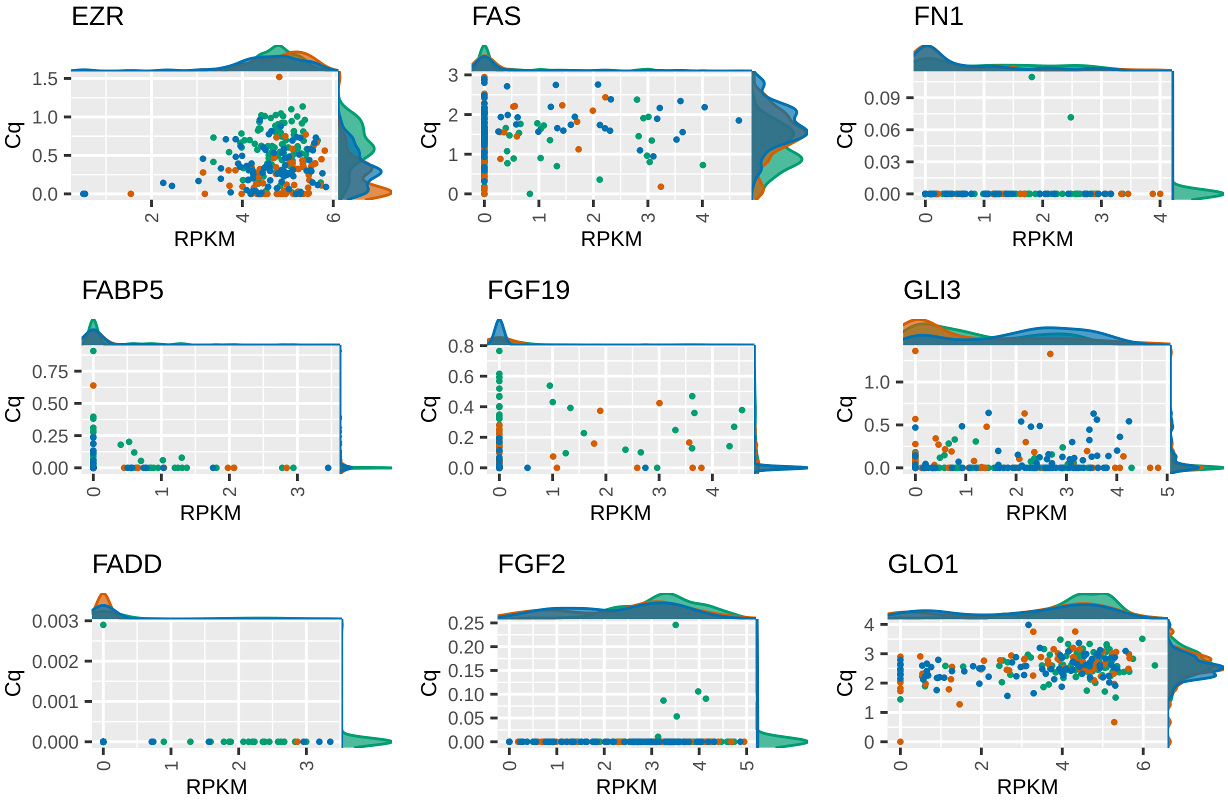

page 5 of 9

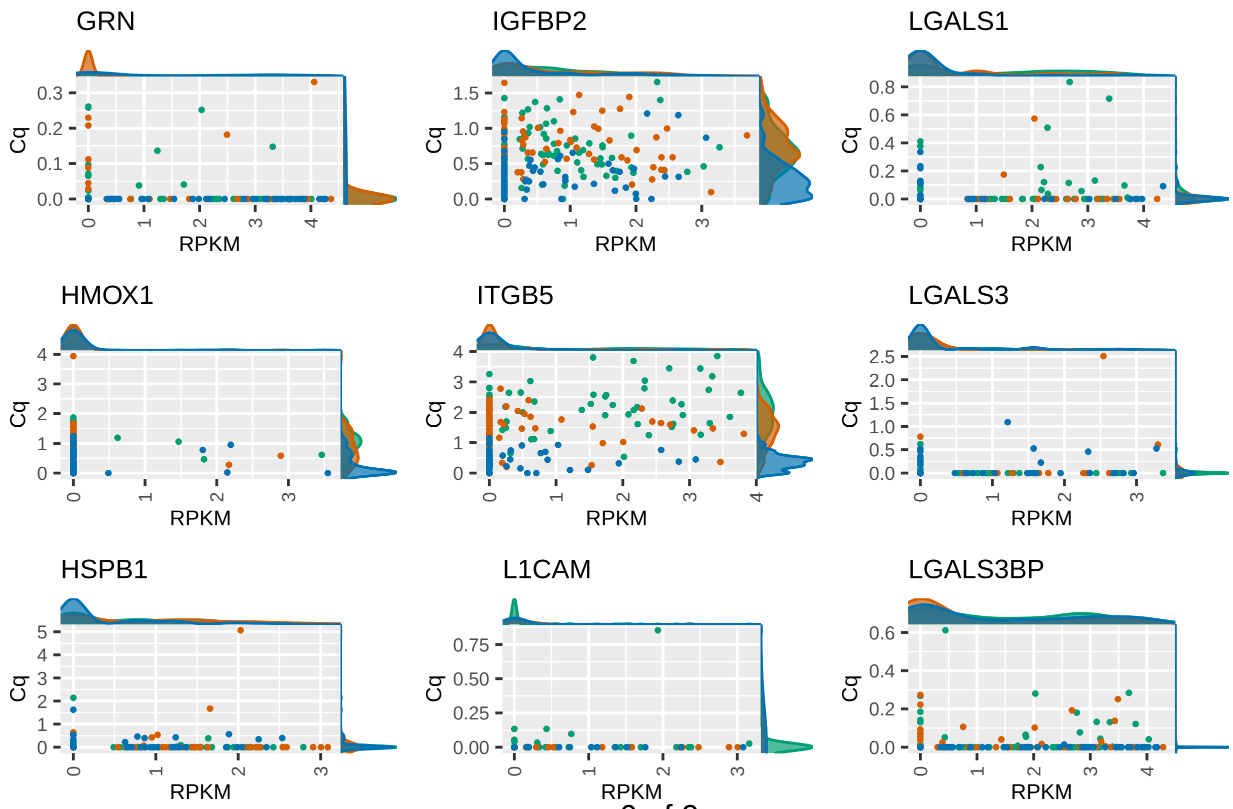

page 6 of 9

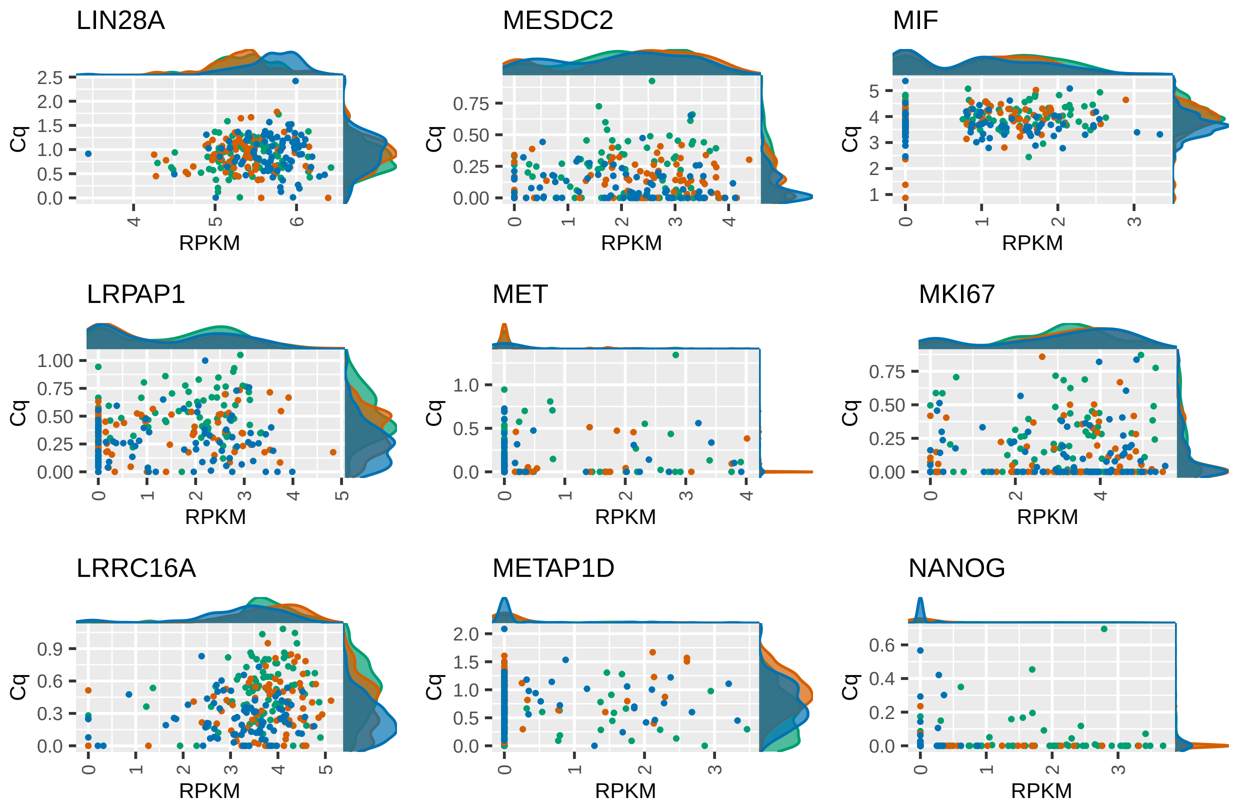

page 7 of 9

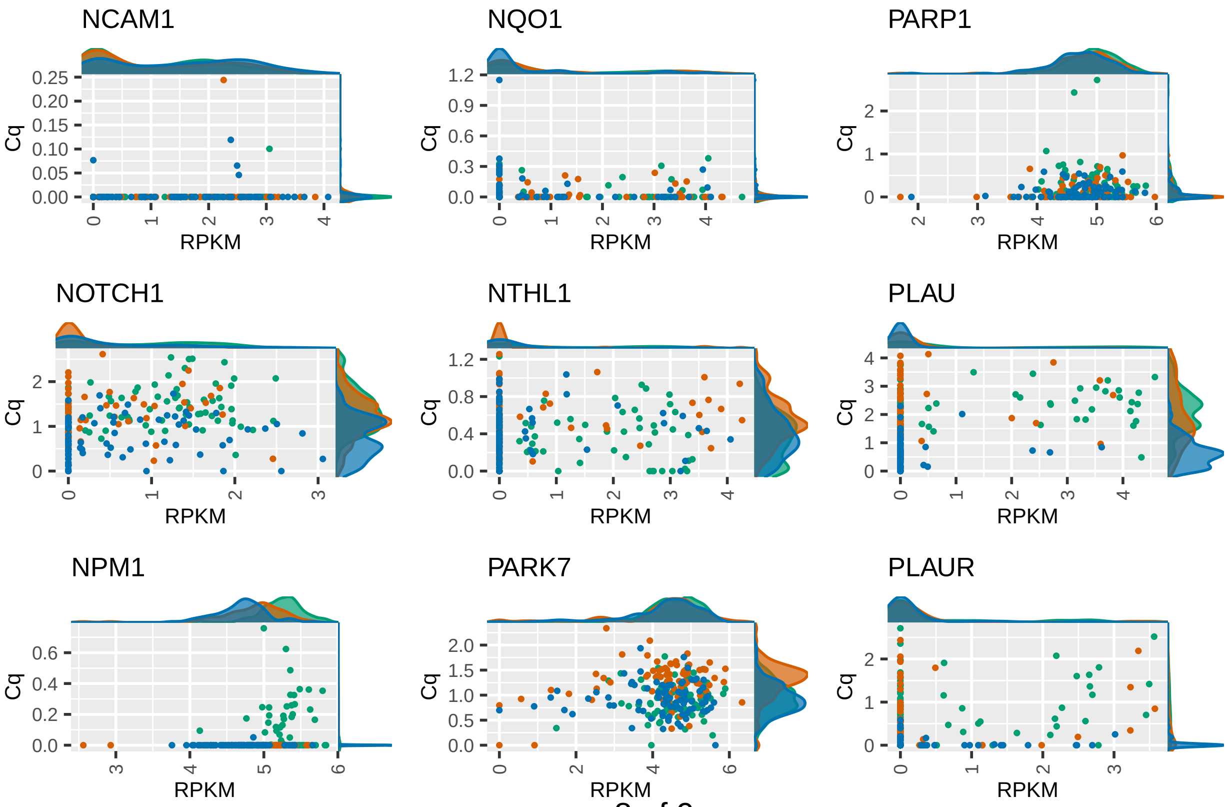

page 8 of 9

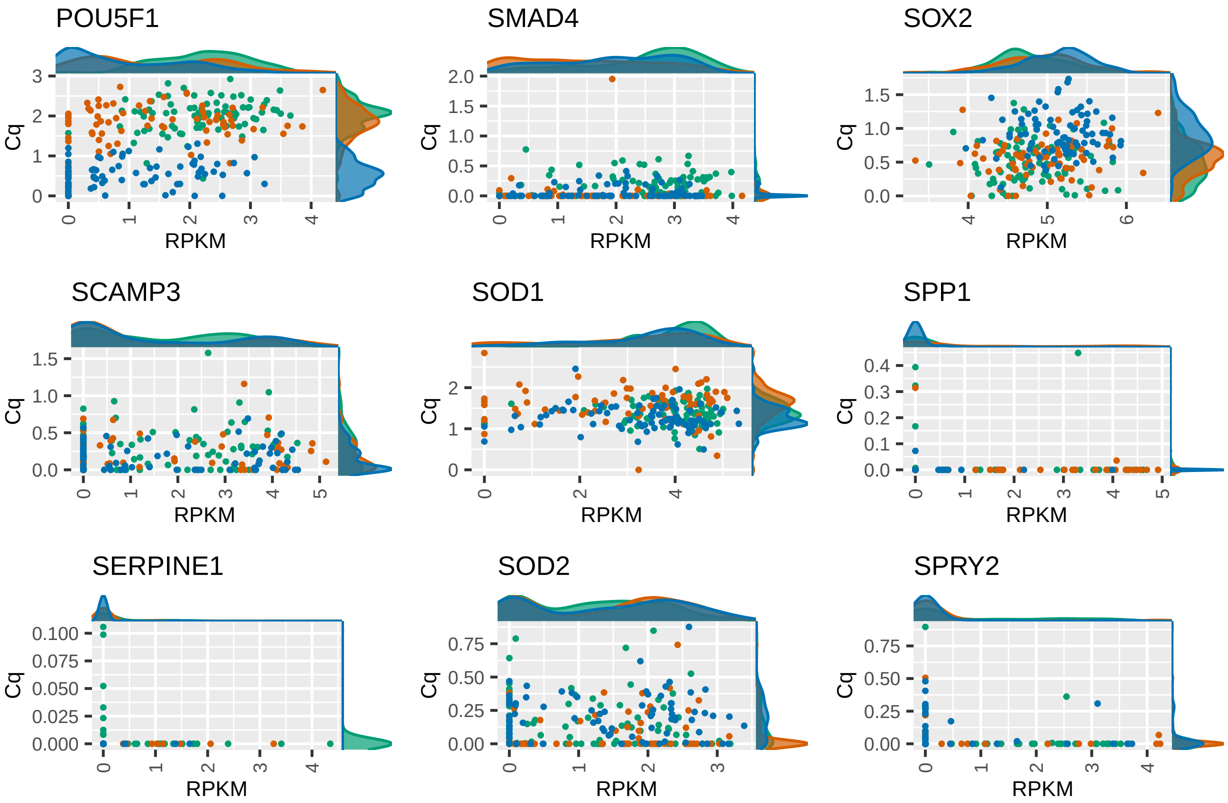

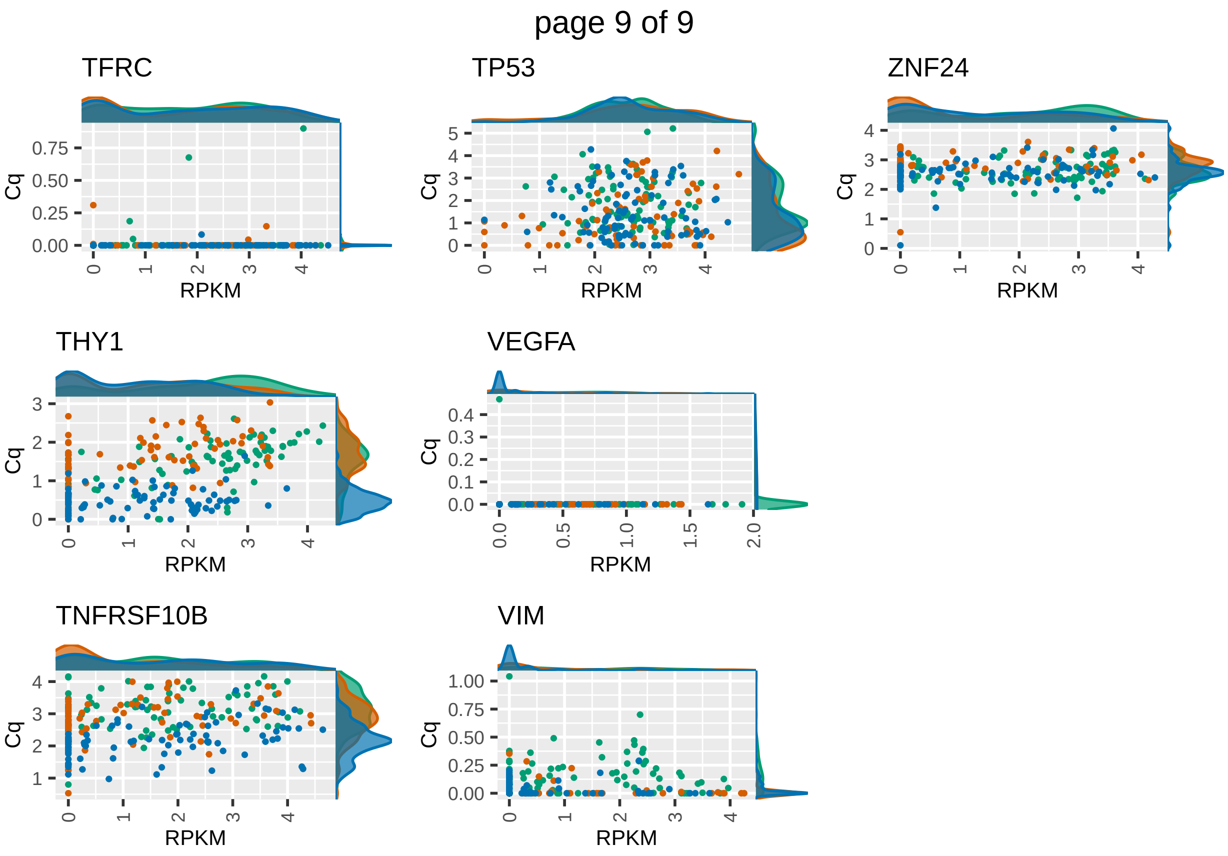

Supplementary Figure 4: RNA and protein gene expression dot and density plots
Combined dot and density plot of mRNA (log RPKM) and protein (Cq) expression in cells measured at 0h (green), 24h (orange) and 48h (blue). The presented data is not normalized for cell cycle, the number of genes detected (RNA) or cumulative protein sums (protein).

Supplementary figure 5

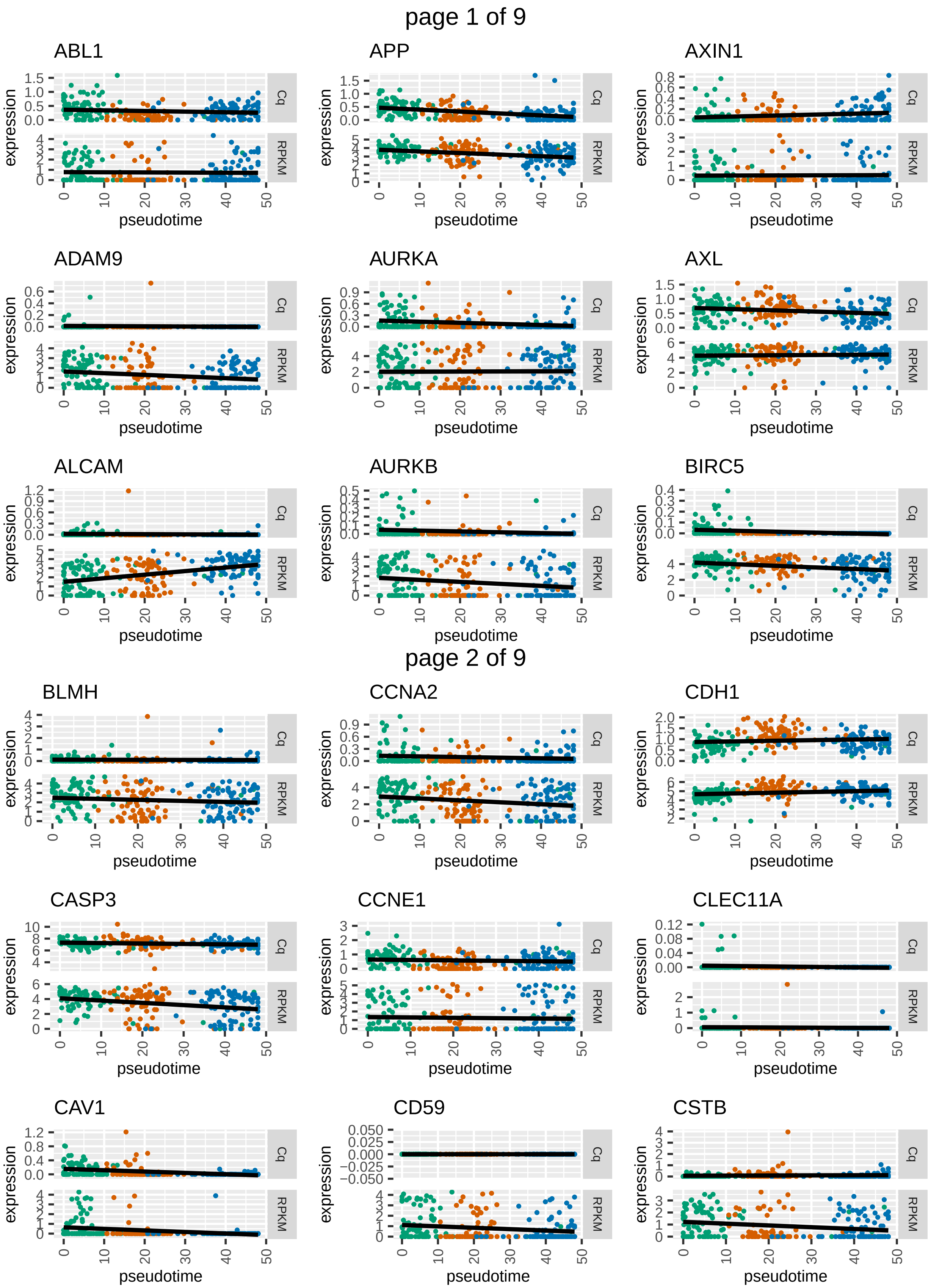

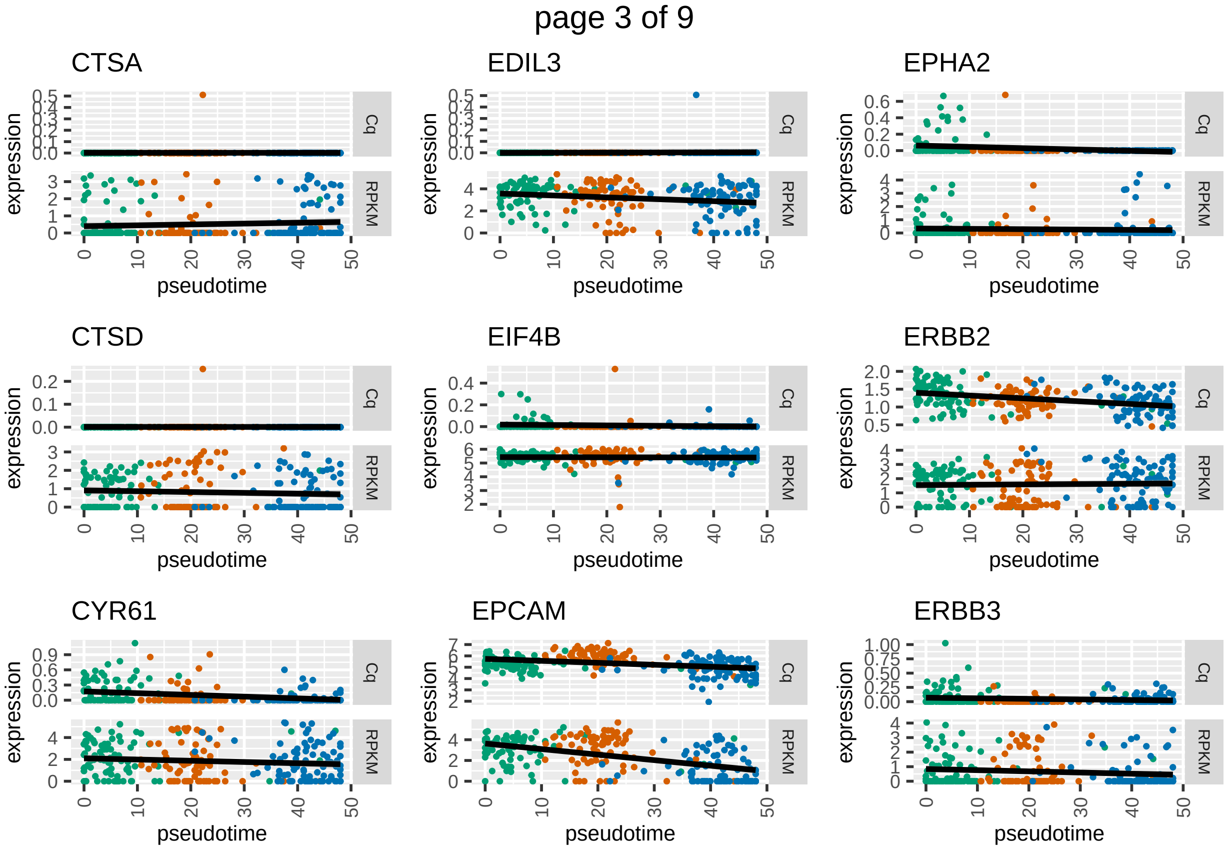
page 3 of 9
CTSA
EDIL3
EPHA2
CTSD
EIF4B
ERBB2
CYR61
EPCAM
ERBB3
expression
pseudotime
Cq
RPKM

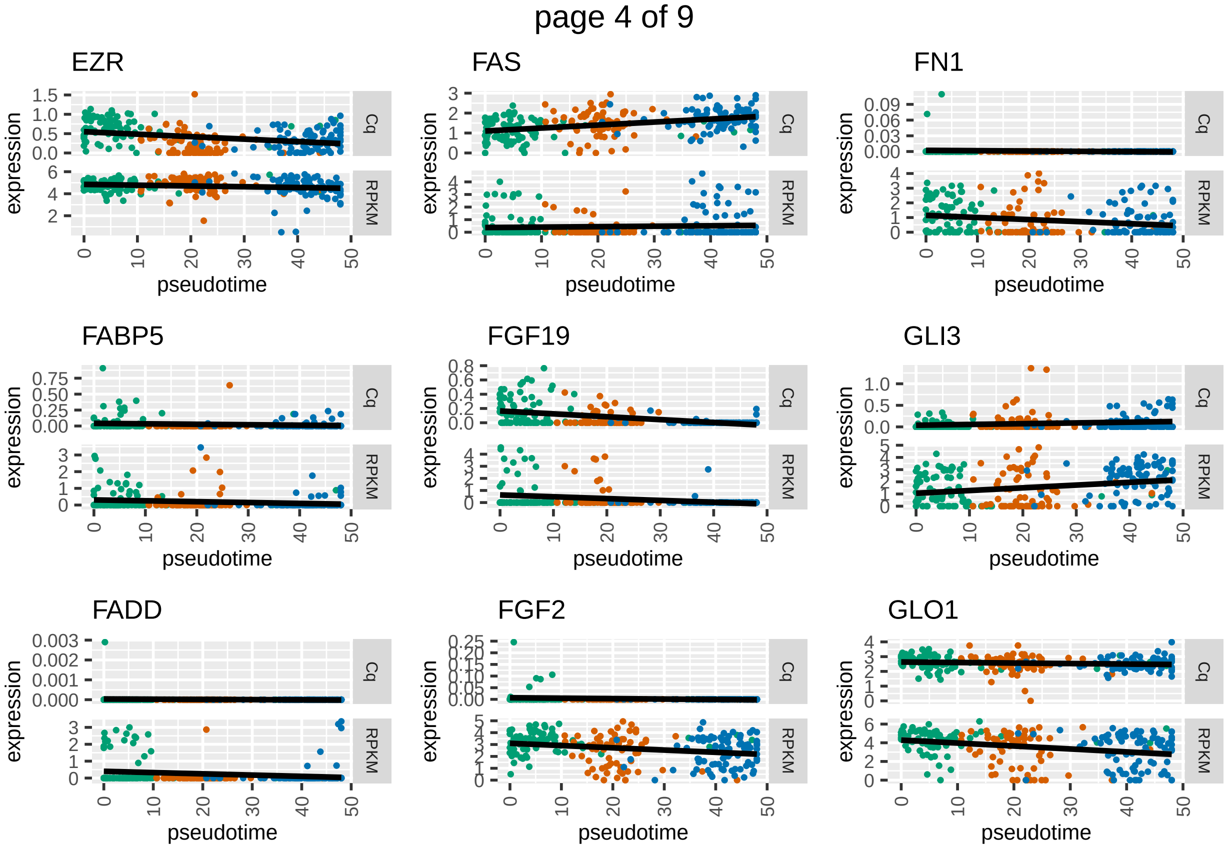
page 4 of 9
EZR
FAS
FN1
FABP5
FGF19
GLI3
FADD
FGF2
GLO1
expression
pseudotime
Cq
RPKM

page 5 of 9

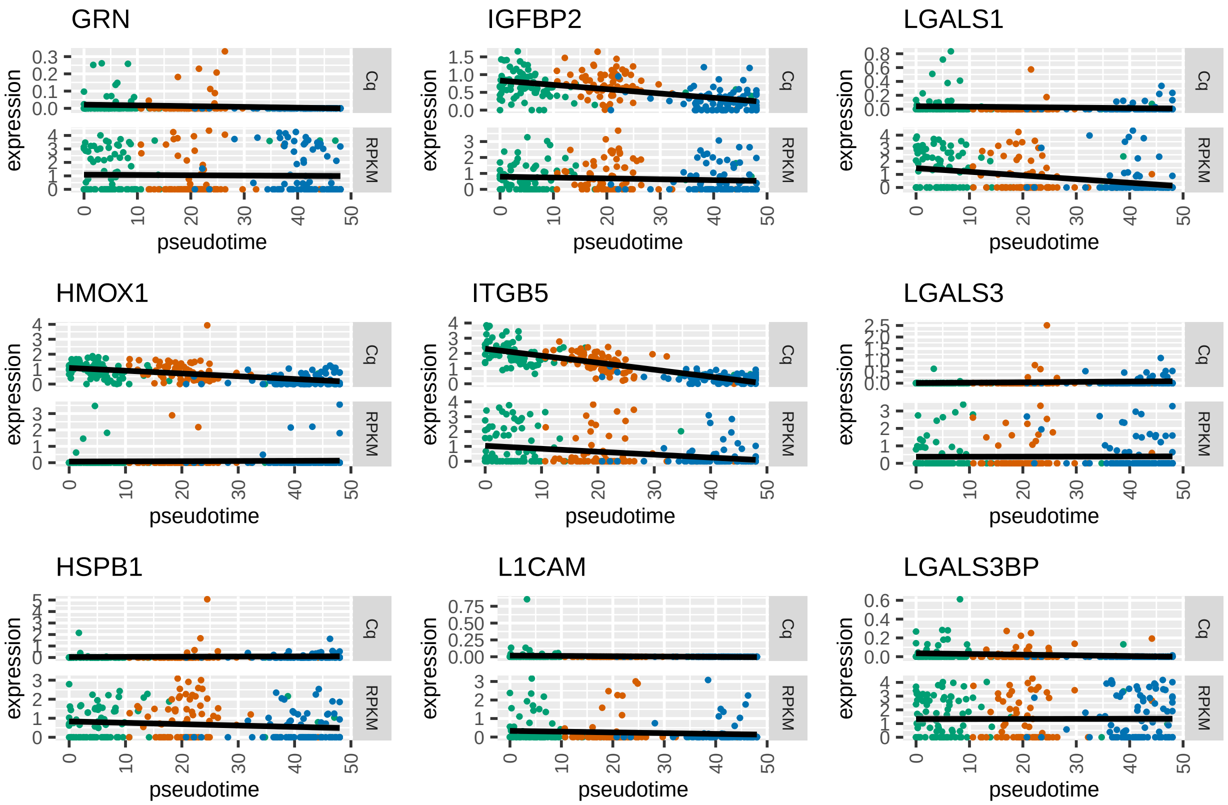

page 6 of 9

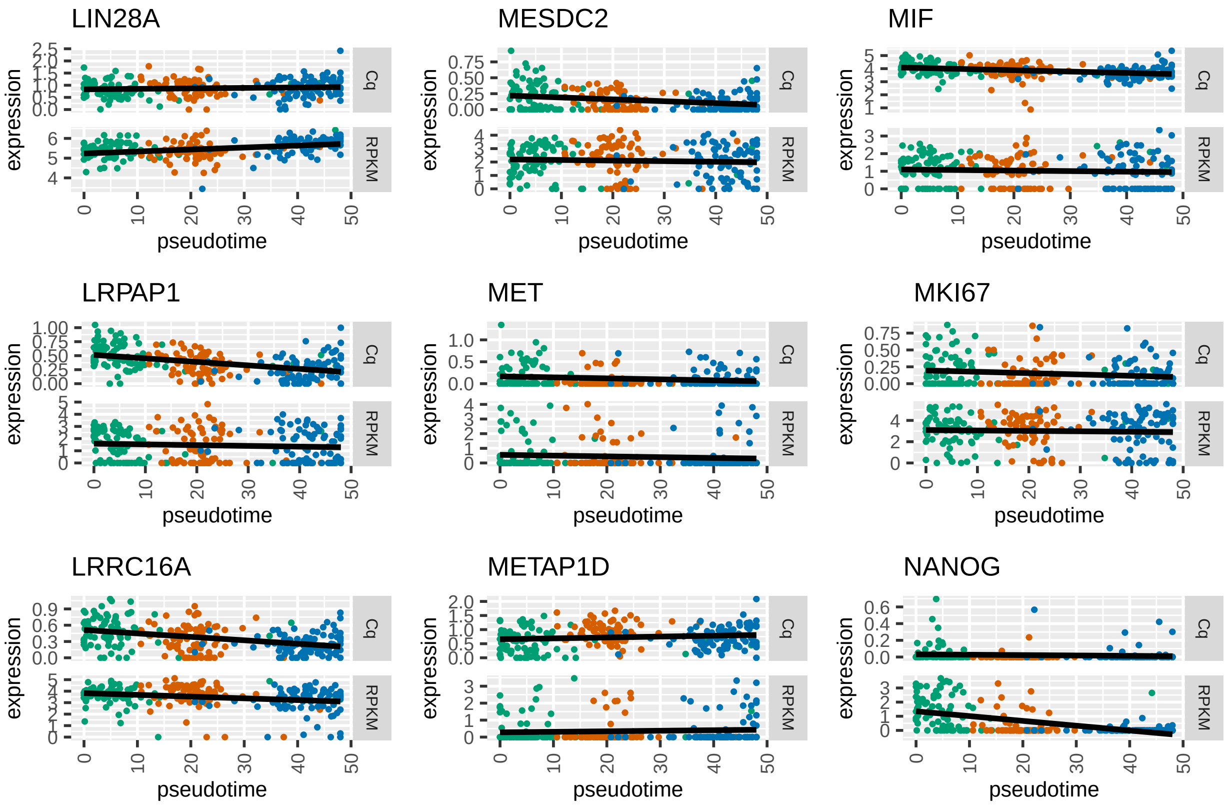

page 7 of 9

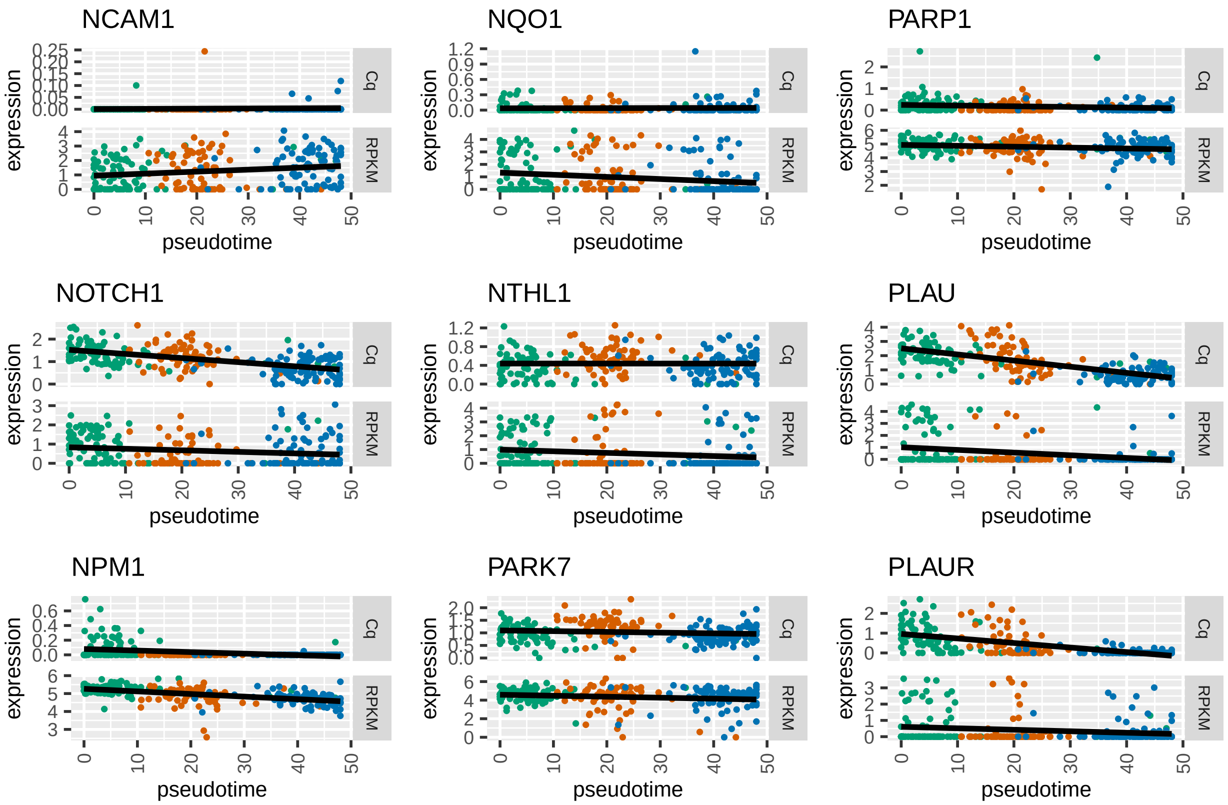

page 8 of 9

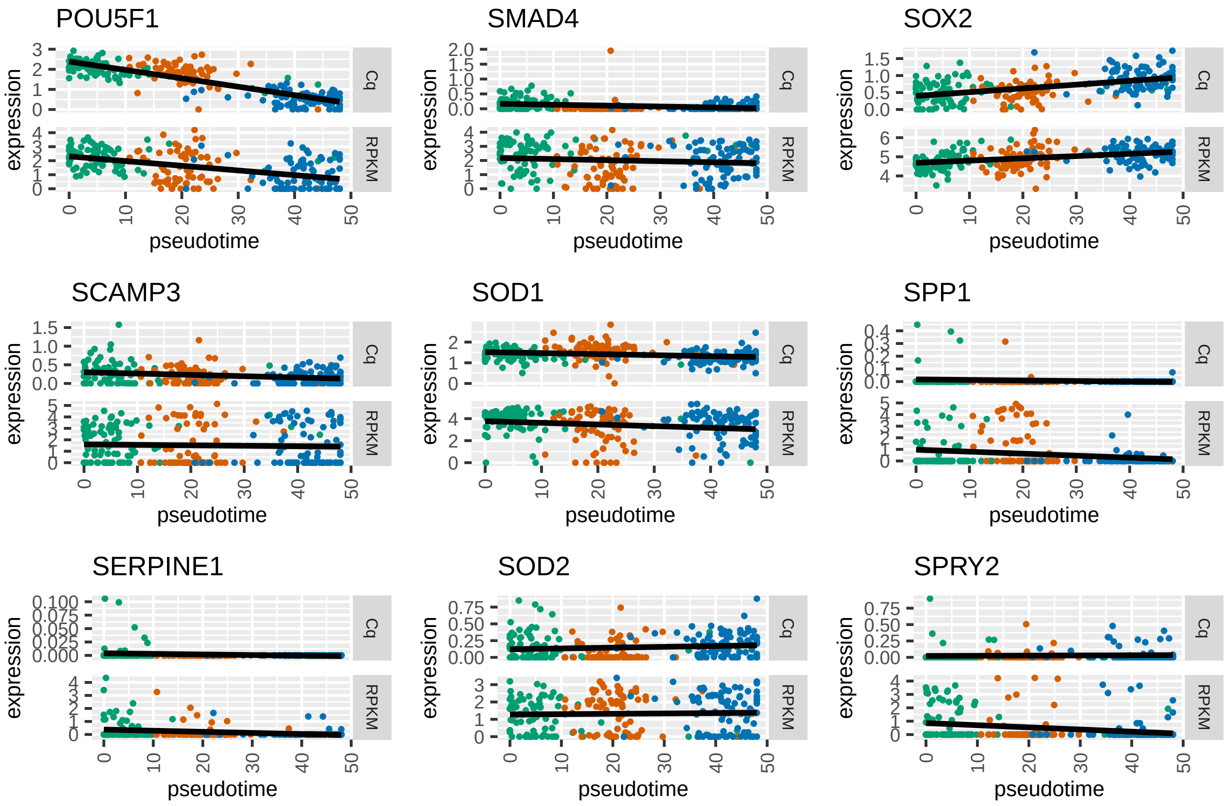

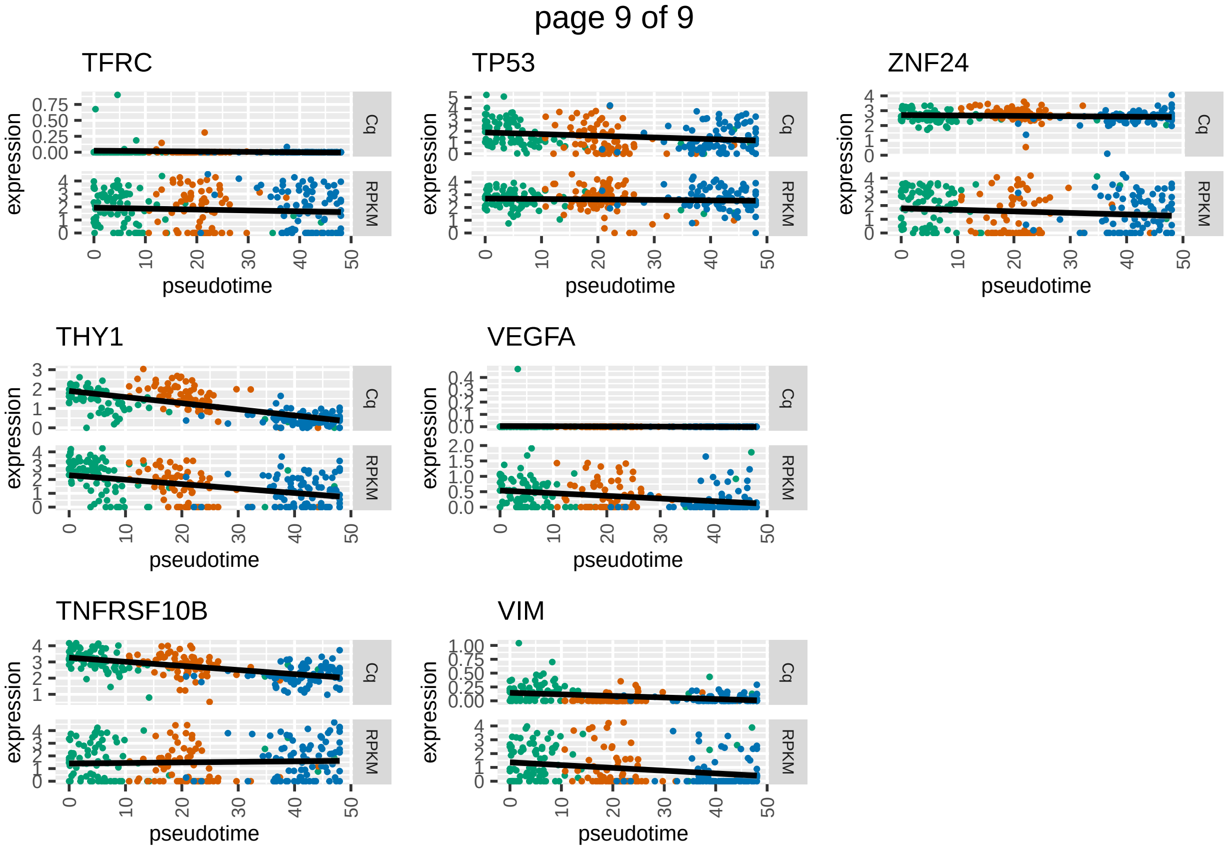

Supplementary Figure 5: RNA and protein gene expression in single cells as ordered by pseudotime mRNA (log RPKM) and protein (Cq) expression in cells ordered by pseudotime as determined by the RNA expression data. The presented data is not normalized for cell cycle, the number of genes detected (RNA) or cumulative protein sums (protein).

Supplementary figure 6

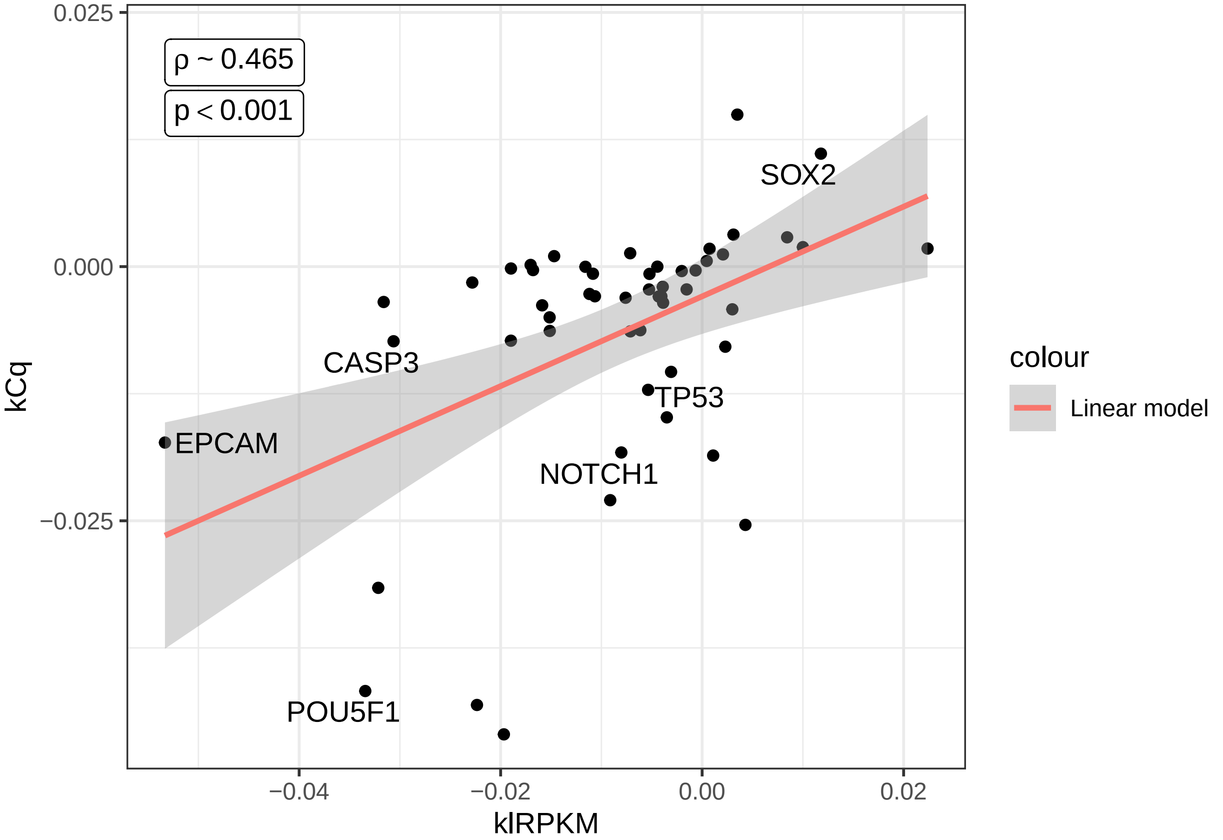

Supplementary Figure 6: Analysis of agreement of mRNA or protein expression changes over time

Agreement of changes of mRNA and protein abundances over the measured time points 0h, 24h and 48h at the level of RNA and protein. The plot shows the results of the linear model Cq(lRPKM)) = 0.49* lRPKM - 0.01 with a Spearman rank correlation coefficient (rho) of 0.48 (p-value 5,1*10-4). Each dot represents a measured gene. The measurements of NOTCH1, POUF51, SOX2, CASP3, EPCAM and TP53 are also identified by their names.

Supplementary figure 7

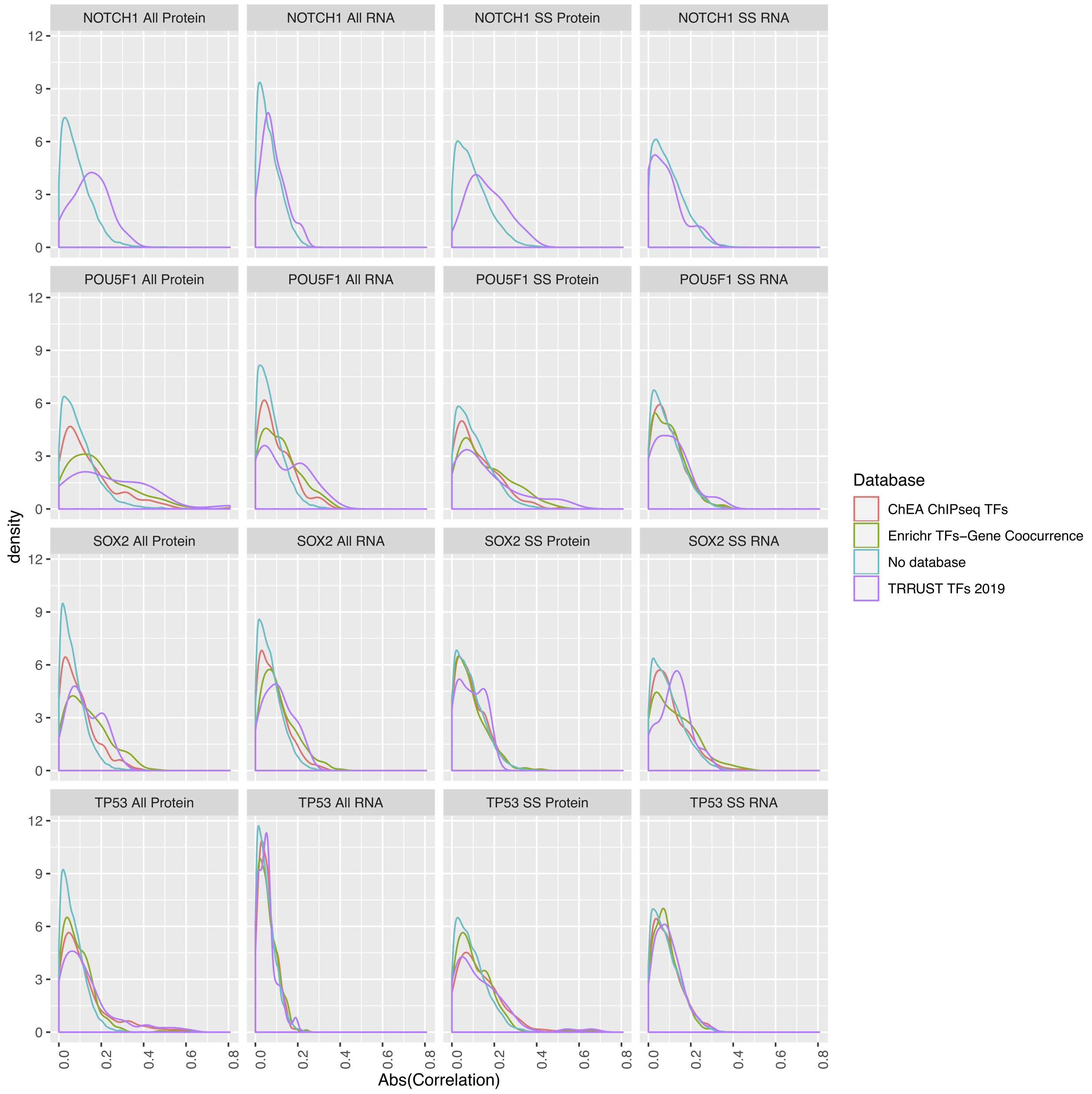

Supplementary Figure 7: Pearson correlation distributions between TFs and known targets

Pearson correlation distribution between transcription factors NOTCH1, POUF51, SOX2 and TP53 and expressed mRNAs. The different distributions are based on if the mRNA are annotated as targets for the TF in three different TF-target databases (**ChEA ChIPseq TFs**, **Enrichr TF-Gene Coocurrence**, **TRRUST TFs 2019**). mRNAs not present in any of the three databases are in the **No database** distribution. The different columns describe what cells that were used, **all** or only cells in steady state(**SS**), and what molecule was used, **protein** or **RNA**, to measure the TF concentration in the cell.

Supplementary figure 8

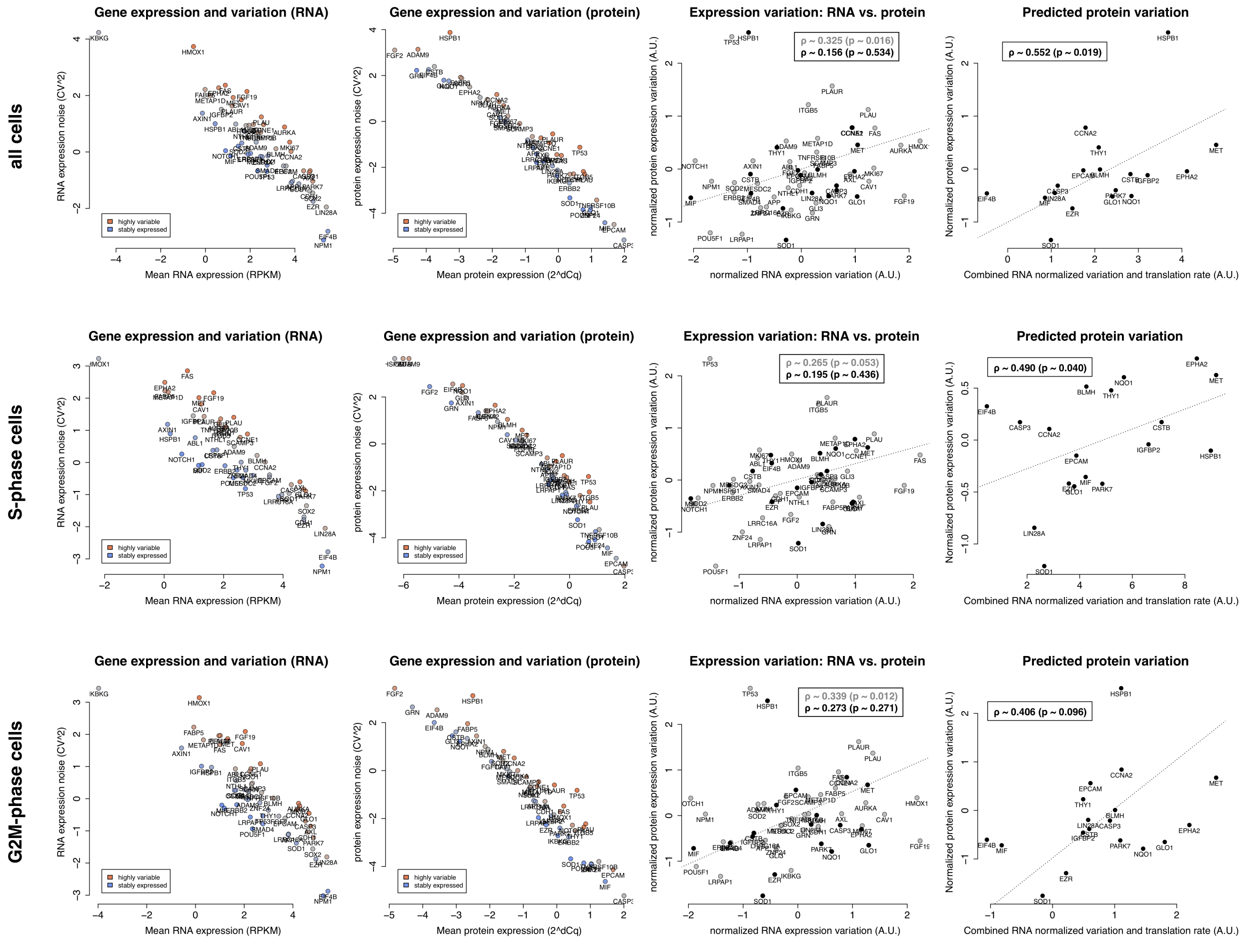

Supplementary Figure 8: Pattern of gene expression variation

Patterns of gene expression variation at steady-state in S-phase cells, G2/M-phase cells and all cells. First column describes the relation between mean RNA expression mean (RPKM) and RNA expression variation (CV2). Colors indicate variably (red) or stably (blue) expressed genes. Second column is similar to first, but for protein. Third column are the normalized gene expression variation (residuals) at the RNA and protein levels. Fourth column are simple additive models of RNA variation and estimated translation rate compared to protein expression variation. Spearman rank correlation coefficient (rho) is given for both columns three and four.

Supplementary tables

Supplementary table 1

Supplementary Table 1

Olink PEA oligo sequence number	Gene name	Uniprot ID
101	FADD	Q13158
102	VEGFA	P15692
103	TNC	P24821
105	IGFBP7	Q16270
106	L1CAM	P32004
107	ANXA1	P04083
108	GLO1	Q04760
109	METAP1D	Q6UB28
110	BLMH	Q13867
111	EZR	P15311
112	EPHA2	P29317
113	FGF2	P09038
114	CSTB	P04080
115	CDH1	P12830
116	GRN	P28799
117	PLAUR	Q03405
118	AXIN1	O15169
120	MKI67	P46013
121	CLEC11A	Q9Y240
122	LGALS3	P17931
123	VIM	P08670
124	NOTCH1	P46531
125	ADAM9	Q13443
126	PARK7	Q99497
127	SERPINE1	P05121
128	FN1	P02751
129	TNFRSF10B	O14763
130	HMOX1	P09601
131	SOD1	P00441
132	CHI3L1	P36222
133	IGFBP2	P18065
134	GLI3	P10071
135	NTHL1	P78549
136	CASP3	P42574
137	HSPB1	P04792
138	POU5F1	Q01860
139	CXCL1	P09341
140	LGALS3BP	Q08380
141	AXL	P30530
142	FGF19	O95750
143	PLAU	P00749
144	FAS	P25445
145	EPCAM	P16422

Supplementary Table 1 *(continued)*

Olink PEA oligo sequence number	Gene name	Uniprot ID
148	TFPI	P10646
149	EDIL3	O43854
150	CXCL8	P10145
151	SCAMP3	O14828
152	CYR61	O00622
153	ERBB2	P04626
154	ERBB3	P21860
155	CCNA2	P20248
156	CTSD	P07339
157	MESDC2	Q14696
158	FABP5	Q01469
159	MIA	Q16674
160	TFRC	P02786
161	ITGB5	P18084
162	SMAD4	Q13485
163	ZNF24	P17028
164	LGALS1	P09382
165	AURKB	Q96GD4
166	MIF	P14174
167	SPRY2	O43597
168	BIRC5	O15392
169	NPM1	P06748
170	ALCAM	Q13740
171	AURKA	O14965
172	TP53	P04637
173	NT5E	P21589
174	LRPAP1	P30533
175	CD59	P13987
176	PARP1	P09874
177	CAV1	Q03135
178	LIN28A	Q9H9Z2
179	EIF4B	P23588
180	FAP	Q12884
181	SOX2	P48431
182	ABL1	P00519
183	MET	P08581
184	SPP1	P10451
185	THY1	P04216
186	VCAM1	P19320
187	LRRC16A	Q5VZK9
188	EGF	P01133
189	NCAM1	P13591
190	NANOG	Q9H9S0
191	CCNE1	P24864
192	IKBKG	Q9Y6K9
193	APP	P05067
194	SOD2	P04179

Supplementary Table 1 *(continued)*

Olink PEA oligo sequence number	Gene name	Uniprot ID
195	NQO1	P15559
196	CTSA	P10619

Supplementary table 2

			K-S test (p-value) *			
			All†		Steady-state‡	
Transcription factor	Database§¶**	Number of targets	Protein	RNA	Protein	RNA
POU5F1	ChEA ChIPseq TFs	261	1.01e-03	**4.06e-04**	**1.63e-01**	4.55e-01
SOX2	ChEA ChIPseq TFs	775	**3.73e-10**	1.49e-04	7.09e-01	**8.82e-02**
TP53	ChEA ChIPseq TFs	319	**1.57e-07**	2.32e-01	**1.02e-06**	9.84e-01
POU5F1	Enrichr TFs-Gene Coocurrence	299	**0.00e+00**	4.18e-13	**5.00e-09**	4.36e-01
SOX2	Enrichr TFs-Gene Coocurrence	299	**0.00e+00**	1.74e-09	3.58e-01	**7.69e-06**
TP53	Enrichr TFs-Gene Coocurrence	299	**2.36e-05**	3.99e-01	**7.18e-03**	1.36e-01
NOTCH1	TRRUST TFs 2019	23	**3.70e-03**	2.72e-01	**1.44e-02**	4.03e-01
POU5F1	TRRUST TFs 2019	51	**2.09e-04**	2.67e-03	**2.64e-01**	4.09e-01
SOX2	TRRUST TFs 2019	50	**1.05e-02**	3.27e-02	7.97e-01	**8.05e-02**
TP53	TRRUST TFs 2019	164	**1.72e-07**	4.84e-01	**6.50e-04**	1.43e-01

* Kolmogorov Smirnov test to compare the distribution of absolute pearson correlation scores of transcription factor to known targets from database to absolute pearson correlation scores of transcription factor to all other genes for the two different molecules (Protein and RNA) and two set of cells (All and Steady-state). The moluecule with the lowest p-value for each of the two sets is highlighted with bold. Distriubiton of scores are visualised in Supplementary figure 8

† All includes all single cells from 0h, 24h and 48h

‡ Steady-State includes all single cells from 0h

§ ChEA: transcription factor regulation inferred from integrating genome wide ChIP X experiments.(PMID: 20709693)

¶ Enrichr: interactive and collaborative HTML5 gene list enrichment analysis tool.(PMID: 23586463 , 27141961)

** TRRUST v2: an expanded reference database of human and mouse transcriptional regulatory interactions.(PMID: 29087512, 26066708)

Supplementary table 3

	Regulatory link weights *†				Pearson correlation‡			
	Protein		RNA		Protein		RNA	
Target	All§	Steady-state¶	All	Steady-state	All	Steady-state	All	Steady-state
AK4	**0.72**	0.70	0.28	0.27	**0.70**	0.62	0.40	0.19
DHRS3	**0.53**	0.46	0.35	0.36	-0.19	0.04	**-0.26**	-0.24
DUSP6	0.53	**0.58**	0.26	0.22	0.31	**0.36**	0.27	0.19
KLHL4	**0.63**	0.55	0.34	0.35	**0.48**	0.24	0.28	0.11
OTX2	**0.68**	0.58	0.37	0.27	**-0.53**	-0.23	-0.28	-0.06
PRR14L	**0.54**	**0.54**	0.35	0.36	**-0.32**	-0.27	-0.13	-0.21
TDGF1	**0.80**	0.72	0.38	0.34	**0.81**	0.52	0.35	0.13
WLS	**0.61**	0.44	0.41	0.32	**-0.41**	-0.10	-0.09	-0.17

* Genie3 gives the weights of the putative regulatory links, with higher weights corresponding to more likely regulatory links

† Bold values are the highest value for the two different molecules (Protein and RNA) and two set of cells (All and Steady-state)

‡ Bold values are the highest positive or negative correlation, dependent if the target is activated or repressed by POU5F1, for the two different molecules (Protein and RNA) and two set of cells (All and Steady-state)

§ All includes all single cells from 0h, 24h and 48h

¶ Steady-State includes all single cells from 0h
