# Supplementary material for: A combined approach for single-cell mRNA and intracellular protein expression analysis
Source: Commun Biol. 2021 May 25;4:624. doi: 10.1038/s42003-021-02142-w (PMC8149646; doi:10.1038/s42003-021-02142-w)
Supplement: Supplementary file 3 — Reporting Summary [file 42003_2021_2142_MOESM3_ESM.pdf]

nature research

Corresponding author(s): Caroline Gallant

Last updated by author(s): March 18, 2021

Reporting Summary

Nature Research wishes to improve the reproducibility of the work that we publish. This form provides structure for consistency and transparency in reporting. For further information on Nature Research policies, see our Editorial Policies and the Editorial Policy Checklist.

Statistics

For all statistical analyses, confirm that the following items are present in the figure legend, table legend, main text, or Methods section.

n/a	Confirmed	
☐	☒	The exact sample size (n) for each experimental group/condition, given as a discrete number and unit of measurement
☐	☒	A statement on whether measurements were taken from distinct samples or whether the same sample was measured repeatedly
☐	☒	The statistical test(s) used AND whether they are one- or two-sided<br>*Only common tests should be described solely by name; describe more complex techniques in the Methods section.*
☐	☒	A description of all covariates tested
☐	☒	A description of any assumptions or corrections, such as tests of normality and adjustment for multiple comparisons
☐	☒	A full description of the statistical parameters including central tendency (e.g. means) or other basic estimates (e.g. regression coefficient) AND variation (e.g. standard deviation) or associated estimates of uncertainty (e.g. confidence intervals)
☐	☒	For null hypothesis testing, the test statistic (e.g. F, t, r) with confidence intervals, effect sizes, degrees of freedom and P value noted<br>*Give P values as exact values whenever suitable.*
☒	☐	For Bayesian analysis, information on the choice of priors and Markov chain Monte Carlo settings
☒	☐	For hierarchical and complex designs, identification of the appropriate level for tests and full reporting of outcomes
☐	☒	Estimates of effect sizes (e.g. Cohen's d, Pearson's r), indicating how they were calculated

Our web collection on statistics for biologists contains articles on many of the points above.

Software and code

Policy information about availability of computer code

Data collection	All processed data used to generate figures are available at Github (https://github.com/b97jre/SPARC).
Data analysis	All code and scripts and data used to generate figures are available at Github (https://github.com/b97jre/SPARC).

For manuscripts utilizing custom algorithms or software that are central to the research but not yet described in published literature, software must be made available to editors and reviewers. We strongly encourage code deposition in a community repository (e.g. GitHub). See the Nature Research guidelines for submitting code & software for further information.

Data

Policy information about availability of data

All manuscripts must include a data availability statement. This statement should provide the following information, where applicable:

- Accession codes, unique identifiers, or web links for publicly available datasets
- A list of figures that have associated raw data
- A description of any restrictions on data availability

The SPARC single-cell sequencing data have been deposited at ENA under project number PRJEB33157.
The SPARC single-cell protein data have been deposited at Scilifelab Data Repository with DOI: 10.17044/scilifelab.14207462
The processed data and code to generate all figures, and all supplementary figures and tables are available at gitihub (b97jre/SPARC) and also been deposited at Scilifelab Data Repository with DOI: 10.17044/scilifelab.14207909

Field-specific reporting

Please select the one below that is the best fit for your research. If you are not sure, read the appropriate sections before making your selection.

- [x] Life sciences
- [ ] Behavioural & social sciences
- [ ] Ecological, evolutionary & environmental sciences

For a reference copy of the document with all sections, see nature.com/documents/nr-reporting-summary-flat.pdf

Life sciences study design

All studies must disclose on these points even when the disclosure is negative.

Sample size	*Describe how sample size was determined, detailing any statistical methods used to predetermine sample size OR if no sample-size calculation was performed, describe how sample sizes were chosen and provide a rationale for why these sample sizes are sufficient.*
Data exclusions	Samples with less than 10 000 reads mapping to the exons or the fraction of spike-in RNAs were greater than 20% were removed from further analysis.
Replication	For each time point and 96-well sorting plate, we sorted approx 88 single cells and (i) population controls of 100 sorted cells/well in duplicate; (ii) buffer, no-cell control in triplicate; (iii) 100 cell equivalent lysate prepared from the SK-MEL-30 cell line. The SK-MEL-30 cell lysate was prepared in bulk, aliquoted and added to every plate in triplicate as an inter-plate control.
Randomization	*Describe how samples/organisms/participants were allocated into experimental groups. If allocation was not random, describe how covariates were controlled OR if this is not relevant to your study, explain why.*
Blinding	*Describe whether the investigators were blinded to group allocation during data collection and/or analysis. If blinding was not possible, describe why OR explain why blinding was not relevant to your study.*

Reporting for specific materials, systems and methods

We require information from authors about some types of materials, experimental systems and methods used in many studies. Here, indicate whether each material, system or method listed is relevant to your study. If you are not sure if a list item applies to your research, read the appropriate section before selecting a response.

Materials & experimental systems

n/a	Involved in the study
[ ]	[x] Antibodies
[ ]	[x] Eukaryotic cell lines
[x]	[ ] Palaeontology and archaeology
[x]	[ ] Animals and other organisms
[x]	[ ] Human research participants
[x]	[ ] Clinical data
[x]	[ ] Dual use research of concern

Methods

n/a	Involved in the study
[x]	[ ] ChIP-seq
[x]	[ ] Flow cytometry
[x]	[ ] MRI-based neuroimaging

Antibodies

Antibodies used	See table S1. DNA-conjugated antibodies were sourced from and validated by Olink Proteomics
Validation	The antibody probes employed in this study were confirmed to detect the expected target (via recombinant proteins and cell lines), and verified no signal was obtained when tested against a large pool of recombinant proteins.

Eukaryotic cell lines

Policy information about cell lines

Cell line source(s)	Human embryonic stem cell line HS181 (hPSCreg KIe001-A)
Authentication	Not authenticated
Mycoplasma contamination	Cell line was negative for mycoplasma infection.
Commonly misidentified lines (See ICLAC register)	*Name any commonly misidentified cell lines used in the study and provide a rationale for their use.*
